# T-bet and RORα control lymph node formation by regulating embryonic innate lymphoid cell differentiation

**DOI:** 10.1038/s41590-021-01029-6

**Published:** 2021-09-23

**Authors:** Christina Stehle, Timo Rückert, Rémi Fiancette, Dominika W. Gajdasik, Claire Willis, Carolin Ulbricht, Pawel Durek, Mir-Farzin Mashreghi, Daniela Finke, Anja Erika Hauser, David R. Withers, Hyun-Dong Chang, Jakob Zimmermann, Chiara Romagnani

**Affiliations:** 1 Innate Immunity, German Rheumatism Research Centre – a Leibniz Institute, Berlin, Germany; 2 Institute of Immunology and Immunotherapy, College of Medical and Dental Sciences, University of Birmingham, Birmingham, B15 2TT, UK; 3 Immune Dynamics, German Rheumatism Research Centre – a Leibniz Institute, Berlin, Germany; 4 Charité – Universitätsmedizin Berlin, corporate member of Freie Universität Berlin and Humboldt-Universität zu Berlin, Department of Rheumatology and Clinical Immunology, Berlin, Germany; 5 Cell Biology, German Rheumatism Research Centre – a Leibniz Institute, Berlin, Germany; 6 Therapeutic Gene Regulation, German Rheumatism Research Centre – a Leibniz Institute, Berlin, Germany; 7 Berlin Institute of Health (BIH) at Charité – Universitätsmedizin Berlin, BIH Center for Regenerative Therapies (BCRT), Charitéplatz 1, 10117 Berlin, Germany; 8 Department of Biomedicine and University Children's Hospital of Basel, University of Basel, Basel, Switzerland; 9 Schwiete Laboratory for Microbiota and Inflammation, German Rheumatism Research Centre – a Leibniz Institute, Berlin, Germany; 10 Department of Cytometry, Institute of Biotechnology, Technische Universität Berlin, Germany; 11 Maurice Müller Laboratories (DBMR), Universitätsklinik für Viszerale Chirurgie und Medizin Inselspital, University of Bern, Bern, Switzerland; 12 Charité – Universitätsmedizin Berlin, corporate member of Freie Universität Berlin and Humboldt-Universität zu Berlin, Department of Gastroenterology, Infectious Diseases, Rheumatology, Berlin, Germany; 13 Leibniz-Science Campus Chronic Inflammation

## Abstract

The generation of lymphoid tissues during embryogenesis relies on group 3 innate lymphoid cells (ILC3) displaying lymphoid tissue inducer (LTi) activity and expressing the master transcription factor RORγt. Accordingly, RORγt-deficient mice lack ILC3 and lymphoid structures, including lymph nodes (LN). Whereas T-bet affects differentiation and functions of ILC3 postnatally, the role of T-bet in regulating fetal ILC3 and LN formation remains completely unknown. Using multiple mouse models and single-cell analyses of fetal ILCs and ILC progenitors (ILCP), here we identify a key role for T-bet during embryogenesis and show that its deficiency rescues LN formation in RORγt-deficient mice. Mechanistically, T-bet deletion skews the differentiation fate of fetal ILCs and promotes the accumulation of PLZF^hi^ ILCP expressing central LTi molecules in a RORα-dependent fashion. Our data unveil an unexpected role for T-bet and RORα during embryonic ILC function and highlight that RORγt is crucial in counteracting the suppressive effects of T-bet.

## Introduction

Innate lymphoid cells (ILCs) comprising group 1 ILCs (ILC1 and NK cells), ILC2 and ILC3 lack rearranged antigen receptors and play an important role in regulating tissue homeostasis and mediating protective responses against pathogens, especially at mucosal surfaces. ILCs develop from the common lymphoid progenitor (CLP) under the influence of IL-2Rγc signalling and their commitment towards the ILC lineage is marked by the up-regulation of a set of genes, including the transcription factor (TF) inhibitor of DNA-binding 2 (Id2), within the ILC progenitor (ILCP)^1^. Among ILC3, α_4_β_7_^+^ CD4^+/-^ lymphoid tissue inducer (LTi) cells are present early in life in fetal liver (FL), spleen, intestine and lymph node (LN) anlage^2,3^, where they interact with mesenchymal stromal cells and guide development of lymphoid structures during embryogenesis^4,5^. LN formation relies on coordinated expression of lymphotoxin (LT) α/β, IL-7 receptor (IL7-R)^6^, Receptor Activator of NF-κB Ligand (RANKL, also known as TRANCE or TNFSF11)^7,8^ and CXCR5^9–11^, which are required for LTi cell attraction, survival and function in LN anlage. CD4^+/-^ LTi cells express the master transcription factor (TF) RORγt, which plays a non-redundant role for their development and thus for the generation of lymphoid tissues, as shown by lack of LN and Peyer’s patches (PP) in RORγt-deficient mice^12,13^. Conversely, the pool of ILC3 expressing RORγt and emerging after birth is preferentially confined to the intestine, with few cells present in LN and spleen. Adult intestinal ILC3 are more diversified and comprise of a subset of CD4^+/-^ CCR6+ LTi-like cells and of CCR6^-^ CD4^-^ ILC3 expressing the natural cytotoxicity receptor NKp46. Generation of NKp46^+^ ILC3 requires up-regulation of ILC1-specifying TF T-bet (encoded by *Tbx21*) and occurs after birth, in conjunction with colonization with Intestinal microbiota^14–19^. Thus, NKp46^+^ ILC3 co-express T-bet and RORγt, along with IL-22 and IFN-γ, and a tightly regulated T-bet/RORγt balance Impacts on the frequency as well as the “NK-ness” of NKp46^+^ ILC3, as shown in *Rorc(gt)*^+/-^, *Tbx21^-/+^* and *Tbx21^-/-^* mice^15,6,20^.

Whereas the antagonistic role of T-bet and RORγt in regulating the balance between ILC3 subsets and ILC1 after birth has been partially elucidated, the interplay between these two TFs during embryonic development, as well as its possible effects on the differentiation of fetal ILCs and on LN formation, has not been investigated and remains completely unknown.

Here, we report that lack of T-bet rescues LN formation in the absence of the ILC3 master TF RORγt. LN formation in mice deficient for both RORγt and T-bet is associated with the accumulation in fetal intestine and LN anlage of a RORα-dependent PLZF^hi^ ILCP population expressing central LTi molecules. These cells are maintained in adult mice, where they produce IL-22 and promote expression of antimicrobial peptides in gut epithelium. Altogether, our data unravel a previously unrecognized role for T-bet and RORα in regulating fetal ILCP differentiation and LN development during embryonic life.

## Results

### scRNA-seq reveals mature ILCs and progenitors in fetal gut

**Extended Data Fig. 1:**
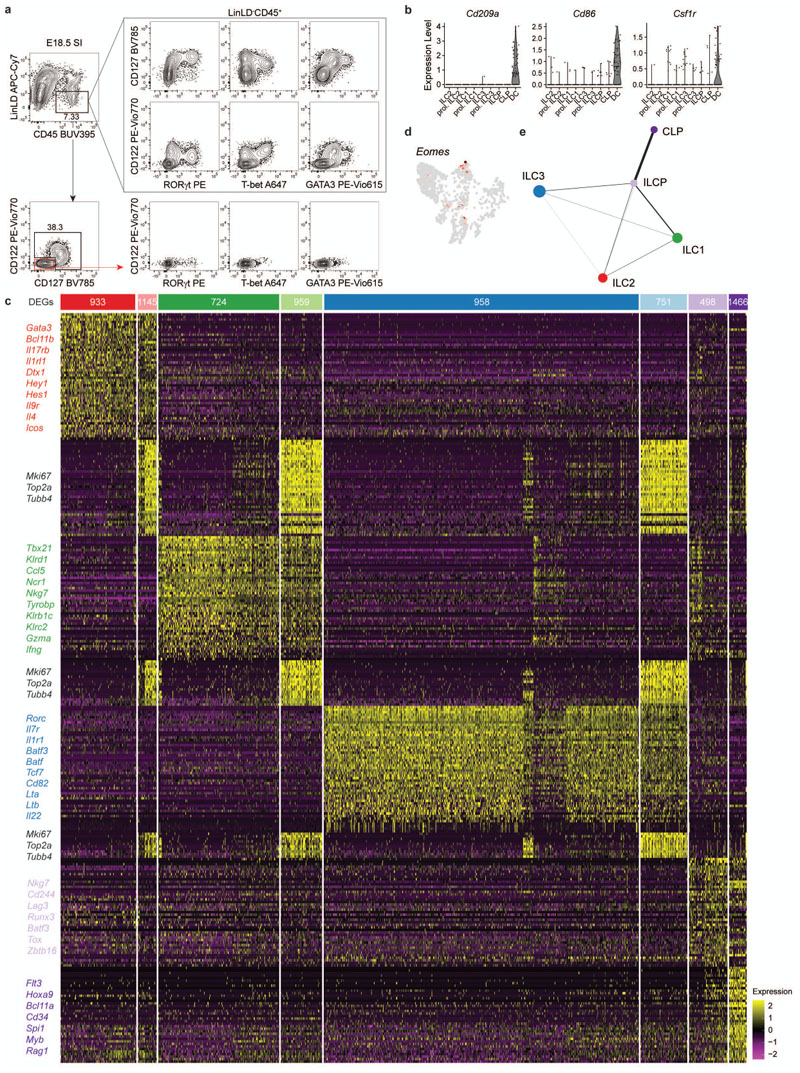
Single-cell sequencing of E18.5 intestinal cells identifies ILC progenitors and mature subsets. **a**, Flow cytometric representative plots showing expression of transcription factors on CD127^+^ and/or CD122^+^ on LinLD^-^CD45^+^ cells of the E18.5 small intestine (SI). **(b)** Violin plots of selected dendritic cell (DC)-associated markers. **(c)** Heatmap displaying the top 50 differentially expressed genes (DEGs) within each cluster. Cluster gene examples are given. **(d)** UMAP-projected expression of Eomes transcripts. **(e)** Trajectory analysis using Partition-based Graph Abstraction (PAGA) lineage interference method.

**Fig. 1:**
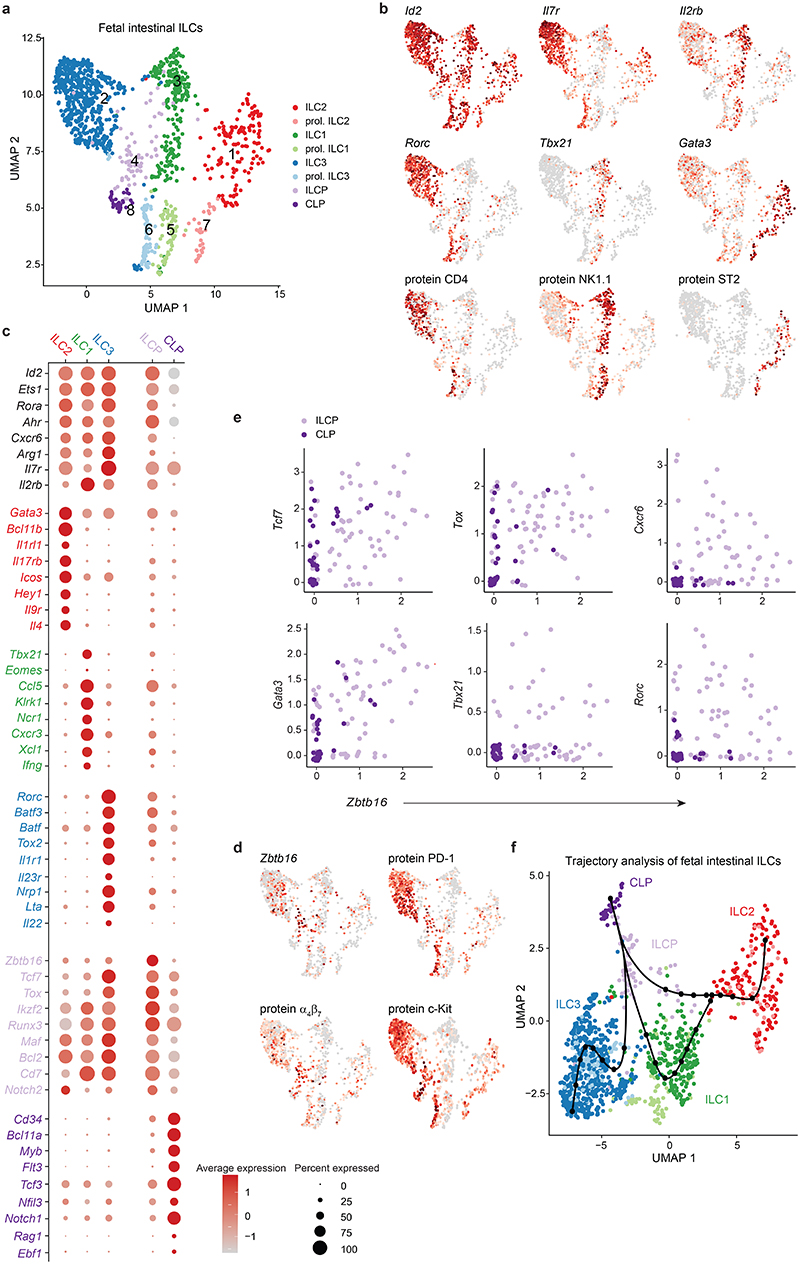
Single-cell sequencing of fetal intestinal cells reveals a spectrum of mature ILC subsets and progenitors. Viable Lin(CD19, CD3, CD5, F4/80, FcɛRIα, Gr-1)^-^ CD45^+^ cells expressing IL-7 receptor (CD127) and/or the IL-2 receptor subunit beta (CD122) isolated from the small intestine (SI) of E18.5 *Rorc(gt)^GFP/wt^* embryos were sort-purified by flow cytometry, and a single-cell expression library was generated using 10x Genomics. **a**, Uniform Manifold Approximation and Projection (UMAP) identifies eight distinct clusters. **(b)** Gene expression and Cellular Indexing of Transcriptomes and Epitopes by Sequencing (CITE-seq) protein expression UMAP plots. **(c)** Selected gene expression within clusters. Colour scale represents average expression, dot size visualizes fraction of cells within the cluster expressing the gene. **(d)** Selected gene expression and CITE-seq protein expression UMAP plots. **(e)** *Zbtb16* co-expression plots of selected genes within CLP and ILCP cluster. **(f)** Trajectory analysis using Slingshot. Inferred trajectories are represented as lines starting within the CLP cluster. Dots represent knots.

In order to study prenatal ILC heterogeneity, we performed single-cell RNA sequencing (scRNA-seq) of viable Lin(CD19, CD3, CD5, F4/80, FcɛRIα, Gr-1)^-^ CD45^+^ cells expressing IL-7 receptor (CD127) and/or the IL-2 receptor subunit beta (CD122) isolated from the small intestine (SI) of embryonic day 18.5 (E18.5) *Rorc(gt)^GFP/wt^* reporter mice. This sorting strategy enabled enrichment of the full spectrum of ILC subsets, including T-bet^+^ ILC1, characterized by low CD127, and high CD122 expression (Extended Data fig. 1a). For simultaneous characterization of RNA and protein, we integrated oligo-conjugated antibodies into the single-cell sequencing workflow, also known as CITE-seq (Cellular Indexing of Transcriptomes and Epitopes by sequencing), which allowed us to validate ILC lineage identities with surface protein expression. After quality control, normalization and filtering out a dendritic cell-like population (displaying transcripts for *Cd209a*, *Cd86* and *Cfs1r*, Extended Data fig. 1b), we analysed 1268 remaining cells with differentially regulated signatures, leading to the identification of eight distinct clusters (Fig. 1a and Extended Data fig. 1c). The common ILC marker Id2 along with expression of *Ets1*, *Rora*, *Ahr*, *Cxcr6* and *Arg1* were detected in the majority of cells belonging to clusters 1-3 and 5-7, assigning them to the ILC lineage (Fig. 1 b,c). Robust gene signatures, including transcripts for *Gata3*, *Tbx21* and *Rorc*, enabled identification of three major superclusters: ILC2 (cluster 1 and 7), ILC1/NK (hereafter referred to as ILC1, cluster 3 and 5) and ILC3/LTi (hereafter referred to as ILC3, cluster 2 and 6). Identity of the inferred superclusters was also confirmed by the distribution of surface markers detected by oligo-conjugated antibodies, ST2, NK1.1 and CD4 (Fig. 1b). Top 50 differentially expressed genes between these clusters included *Bcl11b*, *Il1rl1*, *Il17rb*, *Icos*, *Hey1*, *Il9r* and *Il4* for ILC2; *Ccl5*, *Klrk1*, *Ncr1*, *Cxcr3*, *Xcl1* and *Ifng* for ILC1; *Batf3*, *Batf*, *Tox2*, *Il1r1*, *Il23r*, *Nrp1*, *Lta* and *Il22* for ILC3 (Fig. 1c and Extended Data fig. 1c)^21–23^. Expression of the NK cell TF Eomes was mostly negative and confined to a small subset within *Tbx21^+^* cells, supporting the notion that clusters 3 and 5 largely comprise of ILC1 (Fig. 1c and Extended Data fig. 1d). Transcripts for *Il7r* were highest in ILC3 and lowest in ILC1, which conversely showcased the most prominent expression of *Il2rb* transcripts, as also observed on protein level (Fig 1b,cand Extended Data fig. 1a). Within all ILC superclusters, we further detected clusters of cells (5-7) enriched in gene transcripts associated to active cell cycle, such as *Mki67*, *Top2a* and *Tubb4*, highlighting a substantial fraction of proliferating ILC3, ILC2 and ILC1 within the embryonic intestine (Fig. 1a and Extended Data fig. 1c). Besides identification of classical ILC lineages, two additional clusters (4 and 8) were detected: cluster 8 had little or no expression of most ILC-related genes such as *Id2*, but expressed *Il7r* along with abundant transcripts of *Flt3*, *Cd34*, *Bcl11a* and *Tcf3* (encoding for E2A) (Fig. 1c and Extended Data fig. 1c), indicative of lymphocyte differentiation from hematopoietic stem cells. Moreover, in this cluster we observed few cells expressing recombination activating genes *Rag1/2*, Early B cell Factor 1 (*Ebf1*) and *Nfil3* (Fig. 1c), the latter previously shown to promote early NK/ILC lineage commitment by regulating *Id2*^24–27^. Altogether, this signature was compatible with the one described for common lymphocyte progenitors (CLP)^28^. Cluster 4 shared signatures with ILC precursors (ILCP), formerly identified in FL and adult bone marrow (BM)^17,29–33^. This signature included surface expression of integrin α_4_β_7_ and c-Kit, along with abundant transcripts for *Il7r* and Zbtb16 (encoding for PLZF) (Fig. 1c,d). Accordingly, surface PD-1, which identifies PLZF-expressing ILCP in the BM^32,34^, could be detected within this population and was associated with Zbtb16 transcripts (Figure 1D). Therefore, clusters 8 and 4 are hereafter referred to as CLP and ILCP, respectively. Transition from CLP to ILCP was marked by loss of *CD34*, *Bcl11a* and *Flt3* and up-regulation not only of *Zbtb16*, but also of *Tcf7*, *Tox*, *Ikzf2*, *Runx3*, *Maf*, *Bcl2*, *Cd7*, *Id2*, *Ets1*, *Rora*, *AhR*, *Cxcr6* and *Gata3*, along with a conversion from *Notch1* to *Notch2* expression (Fig. 1c), as previously reported in the BM and FL^35^. Cells within the ILCP cluster largely co-expressed *Zbtb16* with *Tcf7*, *Tox* and *Gata3^lo^*, while few cells showed co-expression of *Zbtb16* with *Cxcr6*, *Tbx21*, *Gata3^hi^* or *Rorc* transcripts (Fig. 1e), suggesting ongoing transition from the ILCP towards the different ILC lineages. Along this line, expression of some ILC1, ILC2 or ILC3-specifying genes such as *Cxcr3*, *Klrk1*, *Xcl1*, *Il17rb*, *Icos*, *Il4*, *Batf3*, *Batf*, *Nrp1* and *Il1r1* could be accordingly observed within the ILCP cluster (Fig. 1c and Extended Data fig. 1c). To model continuous developmental trajectories, we regressed out cell-cycle related genes within the proliferating clusters (5, 6, 7), to map these cells onto their respective ILC superclusters and applied two distinct computational models of single-cell trajectory inference, Slingshot (Fig. 1f) and partition-based graph abstraction (PAGA) (Extended Data fig. 1e). Both methods inferred comparable trajectories with continuous cell transitions from CLP to ILCP, further branching into ILC1, ILC2 and ILC3 (Fig. 1f and Extended Data fig. 1e). Interestingly, Slingshot analysis highlighted an early branching of the ILCP towards the ILC2 cluster, while ILC1 and ILC3 retained a common trajectory and transitional states before bifurcating into separate lineages (Fig. 1f).

Altogether, these data report transcriptional signatures of ILC lineages in the embryonic SI and identify a spectrum of progenitor populations within the isolated tissue that resemble those previously described in FL or adult BM, suggesting the intestine as an active ILC differentiation niche during embryonic development.

### Fetal intestinal ILC3 are heterogeneous

**Extended Data Fig. 2:**
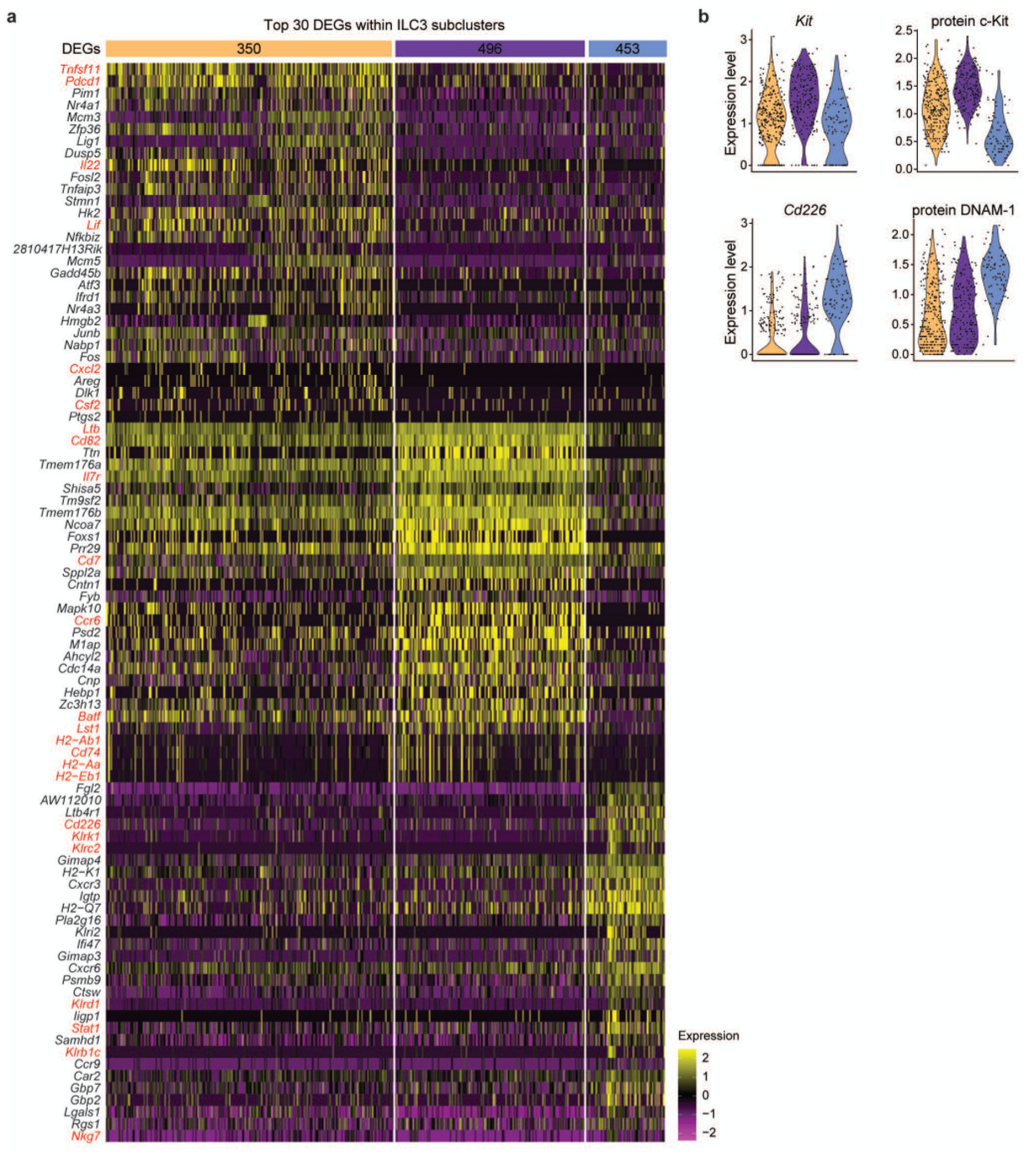
Gene expression of specified ILC3 subsets defined by scRNA-seq. **a**, Heatmap displaying the top 30 differentially expressed genes (DEGs) and **(b)** expression levels of selected genes and corresponding proteins as assessed by CITE-seq within ILC3 subclusters.

**Fig. 2:**
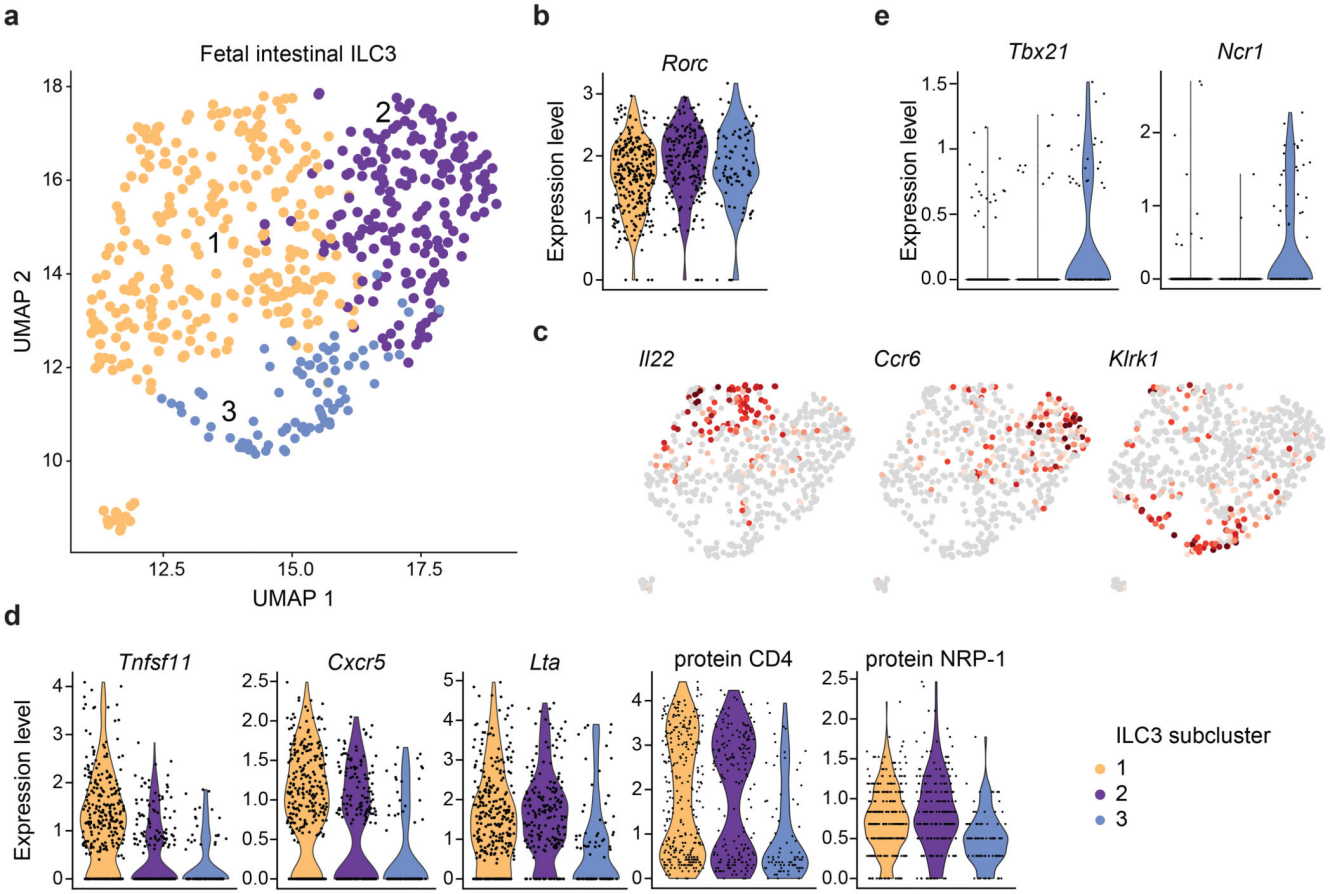
Transcriptomic profiling on single-cell level discloses heterogeneity within the embryonic ILC3 supercluster. **a,** UMAP dimensional reduction projection reveals three separate subclusters within the ILC3 cluster. **(b)** Violin plot of *Rorc* expression within ILC3 subclusters. **(c)** Selected gene expression UMAP plots. **(d)** Violin plots of expressed transcripts or proteins within ILC3 subclusters. **(e)** Violin plots of *Tbx21* and *Ncr1* within ILC3 subclusters.

In order to assess whether ILC3 from fetal SI comprise heterogeneous subsets similar to their adult counterparts, we next analysed the ILC3 supercluster in more detail (Figure 2). Using unsupervised clustering, we identified three subclusters with defined sets of differentially regulated genes, displaying analogous *Rorc* expression (Fig. 2a,b). Among the top 30 genes characterizing subcluster 1, we found *Tnfsf11* (encoding RANKL or TRANCE), *Pdcd1* and transcripts for cytokines or chemokines such as *Il22*, *Lif^36^*, *Csf2* (encoding GM-CSF) and the neutrophil chemoattractant *Cxcl2* (Fig. 2c,dand Extended Data fig. 2a). In addition, *Cxcr5* transcripts were also enriched in subcluster 1 (Fig. 2d). Subcluster 2 showed high expression of *Il7r*, *Ltb*, *Ccr6*, *Batf* and *Cd7* (Fig. 2c and Extended Data fig. 2a). Transcripts associated with major histocompatibility (MHC) class molecules, including H2-genes as well as *Cd74*, *Cd82* and *Lst1*, previously reported in CD4^+^ fetal LTi cells and in CCR6^+^ ILC3 postnatally,^6,21,37–40^, were largely confined to subcluster 2 (Extended Data fig. 2a). Subcluster 3 was characterized by low expression of c-Kit (protein and transcripts) (Extended Data fig. 2b) and high expression of genes typically associated to ILC1 and NK, as well as adult NKp46^+^ ILC3, such as *Klrk1*, *Klrc2*, *Klrd1*, *Klrb1c* together with *Cd226* transcripts and surface protein (Fig. 2c and Extended Data fig. 2a,b). Importantly, we also detected cells expressing transcripts for *Tbx21* and *Ncr1* (Fig. 2e). Notably, expression of *Lta* transcripts and of surface proteins CD4 and NRP-1 were comparable in subcluster 1 and 2, although also present at low levels in subcluster 3 (Fig. 2d). These data suggest that generation of specified ILC3 subsets already happens embryonically and point out that a small subset of T-bet^+^ ILC3 is present prenatally.

### RORγt^+^T-bet^+^CD4^+^ ILC3 transiently emerge prenatally

**Extended Data Fig. 3:**
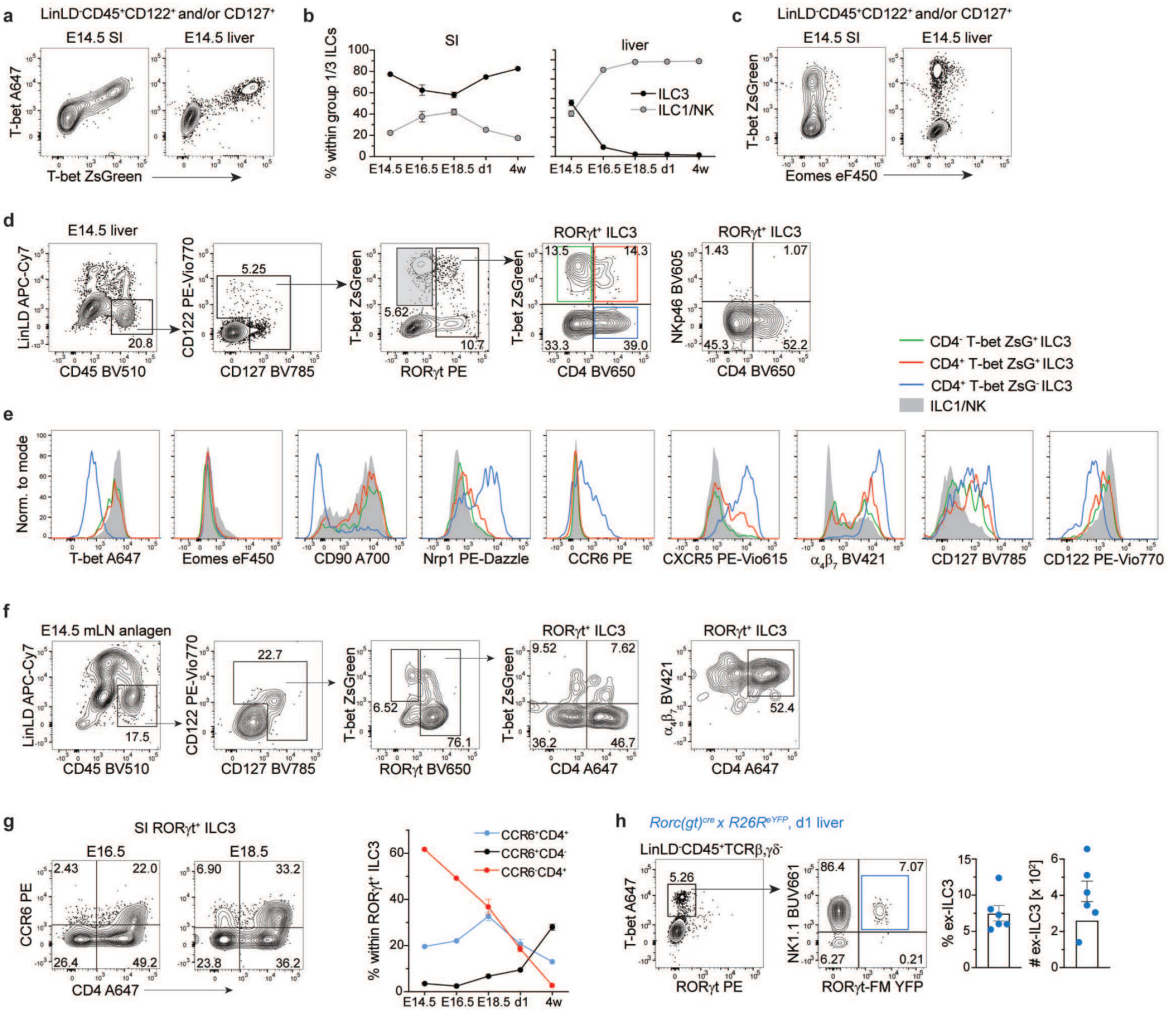
Gene expression of specified ILC3 subsets defined by scRNA-seq. **a**, Heatmap displaying the top 30 differentially expressed genes (DEGs) and **(b)** expression levels of selected genes and corresponding proteins as assessed by CITE-seq within ILC3 subclusters.

**Fig. 3:**
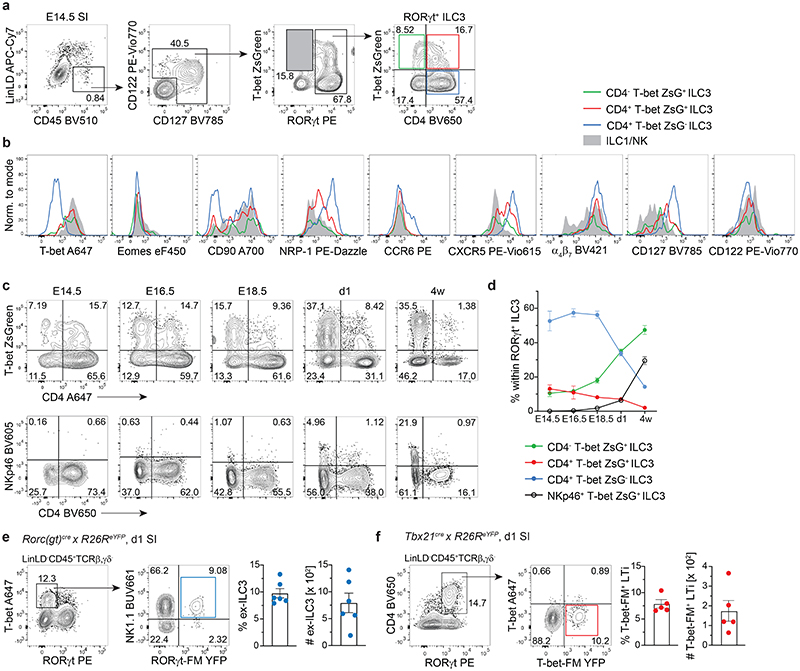
A subset of RORγt^+^T-bet^+^CD4^+^ ILC3 emerges during embryonic development. **(a-d)** Flow cytometric analysis of embryonic T-bet ZsGreen reporter mice. a, Representative gating of E14.5 small intestine (SI) for identification of T-bet^+^RORγt^-^ ILC1 (grey shaded gate) and RORγt^+^ ILC3 subsets based on expression of CD4 and T-bet ZsGreen. **(b)** Representative histograms of marker expression by ILC1/NK or ILC3 subsets from E14.5 SI. **(c)** Representative plots of T-bet ZsGreen or NKp46 and CD4 expression by RORγt^+^ ILC3 isolated from SI at different indicated embryonic and postnatal time-points. **(d)** Quantification of frequency of designated RORγt^+^ ILC3 subsets over time in ontogeny. Graphs depict data as mean ± SEM, E14.5 n=4 (pooled samples) examined over 4 independent experiments, E16.5 n=2 (pooled samples) examined over 2 independent experiments, E18.5 n=10 (individual samples) examined over 3 independent experiments, d1 n=8 (individual samples) examined over 2 independent experiments, 4 w n=7 examined over 2 independent experiments. **(e)** Representative SI gating on T-bet^+^ RORγt-protein^-^ ILC1 and identification of NK1.1^+^RORγt-fate map(FM)^+^ ex-ILC3 in one day old *Rorc(gt)*^cre/wt^ × *R26^eYFP^* newborn mice. Quantification of frequencies and absolute numbers of ex-ILC3 as mean ± SEM, n=6 examined over 2 independent experiments. **(f)** Representative SI gating on RORγt-protein^+^CD4^+^ LTi cells and identification of T-bet^-^protein^-^T-bet^-^FM^+^ ILC3 in one day old *Tbx21^cre/wt^ × R26^eYFP^* newborn mice. Quantification of frequencies and absolute numbers of T-bet-FM^+^ LTi cells as mean ± SEM, n=5 examined over 2 independent experiments. Detailed statistics are available in source data.

In order to validate our scRNA-seq data by flow cytometry and to understand when T-bet^+^ ILC3 emerge during embryonic development, we took advantage of T-bet-ZsGreen reporter mice and performed a kinetic analysis of T-bet and RORγt expression by LinLD^-^ CD45^+^CD122^+^ and/or CD127^+^ cells derived from the developing small intestine (SI) or FL. T-bet-ZsGreen reporter signal was validated by intracellular staining of T-bet, showing full co-expression of T-bet and ZsGreen fluorescent proteins (Extended Data fig. 3a). Both ILC3 and ILC1 were present in the SI of E14.5 mice, with ILC3 persistently dominating over ILC1 throughout embryonic development until 4 weeks of age (Fig. 3a and Extended Data fig. 3b). Eomes was only expressed on a small subset of T-bet^+^ cells (Fig. 3 b and Extended Data fig. 3c and e), in line with E18.5 scRNA-seq data. Although percentage of LinLD-CD45^+^CD122^+^ and/or CD127^+^ cells was lower in FL compared to SI (Fig. 3a and Extended Data fig. 3d), ILC3 and ILC1 could be observed at similar frequency in FL at E14.5, with percentage of ILC3 dropping around E16.5, concomitant with ILC1 increase (Extended Data fig. 3b). By zooming Into RORγt+ ILC3 we observed that, besides CD4^+^ T-bet^-^ cells, around 25% of E14.5 intestinal or FL ILC3 expressed T-bet, and a significant fraction co-expressed CD4 (Fig. 3a and Extended Data fig. 3d). A similar distribution of ILC3 subsets according to T-bet and CD4 was also observed in the E14.5 mesenteric LN (mLN) anlage (Extended Data fig. 3f). Further characterization of intestinal and liver ILC3 subsets showed high surface expression of CD127, NRP-1 and *CXCR5* in the CD4^+^T-bet^-^ subset, with intermediate levels in CD4^+^T-bet^+^ and lowest expression in CD4^-^T-bet^+^ cells. All ILC3 subsets expressed high levels of α_4_β_7_; conversely, CD90 was enriched in T-bet^+^ compared to T-bet^-^ ILC3 (Fig. 3b and Extended Data fig. 3e). Over time, the percentage of CD4^-^T-bet^+^ ILC3 increased gradually in the intestine, while CD4^+^T-bet^-^ and CD4^+^T-bet^+^ ILC3 decreased from E14.5 to 4 weeks of age (Fig. 3, c and d). Notably, regardless of T-bet expression, NKp46 was not detected on the surface of ILC3 during embryonic development and appeared only in newborn mice (Fig. 3c), in line with previous findings^14^. CCR6 was selectively expressed by a small fraction of intestinal CD4^+^T-bet^-^ ILC3 cells by E14.5 (Fig. 3b), while its expression increased until E18.5, when CCR6^+^CD4^-^ ILC3 first appeared (Extended Data fig. 3g). Over time, there was a reduction in the frequency of CCR6-CD4^+^ ILC3, which were virtually absent postnatally, when CCR6^+^CD4^+/-^ became the predominant ILC3 populations (Extended Data fig. 3g).

Our data showed that CD4^+^Tbet^+^ cells represented a consistent proportion of intestinal ILC3 at E14.5, in contrast to 4-week-old mice. As their frequency was already strongly reduced at birth, we analysed newborn fate map *Rorc(gt)^cre^ × R26R^eYFP^* (RORγt-FM) or *Tbx21^cre^ × R26R^eYFP^* (T-bet^-^FM) reporter mice to test whether CD4^+^ T-bet^+^ ILC3 might have undergone lineage reprogramming already during embryonic life. Indeed, a population of “ex-ILC3” which has switched off expression of RORγt, likely in response to inflammatory signals such as IL-12, has been previously described in the adult intestine^15,17,41–43^. Among T-bet^+^ RORγt^-^ cells, we found a population of NK1.1^+^ RORγt-FM^+^ ILC1 in both liver and intestine, showing that ex-ILC3 are not only generated postnatally in response to environmental or inflammatory signals, but are already present at birth (Fig. 3e and Extended Data fig. 3h). Reciprocally, we also detected a small but consistent fraction of T-bet^-^FM^+^ cells among RORγt^+^CD4^^+^^ ILC3 in the intestine of newborn mice (Fig. 3f), suggesting a history of T-bet expression in these cells.

In summary, we found that T-bet is expressed by subsets of intestinal ILC3 already during embryonic development and identified a population of T-bet^+^CD4^+^ ILC3, which progressively decrease over time, revealing previously unappreciated prenatal ILC plasticity.

### Lack of T-bet rescues formation of LN in RORγt-deficient mice

**Extended Data Fig. 4:**
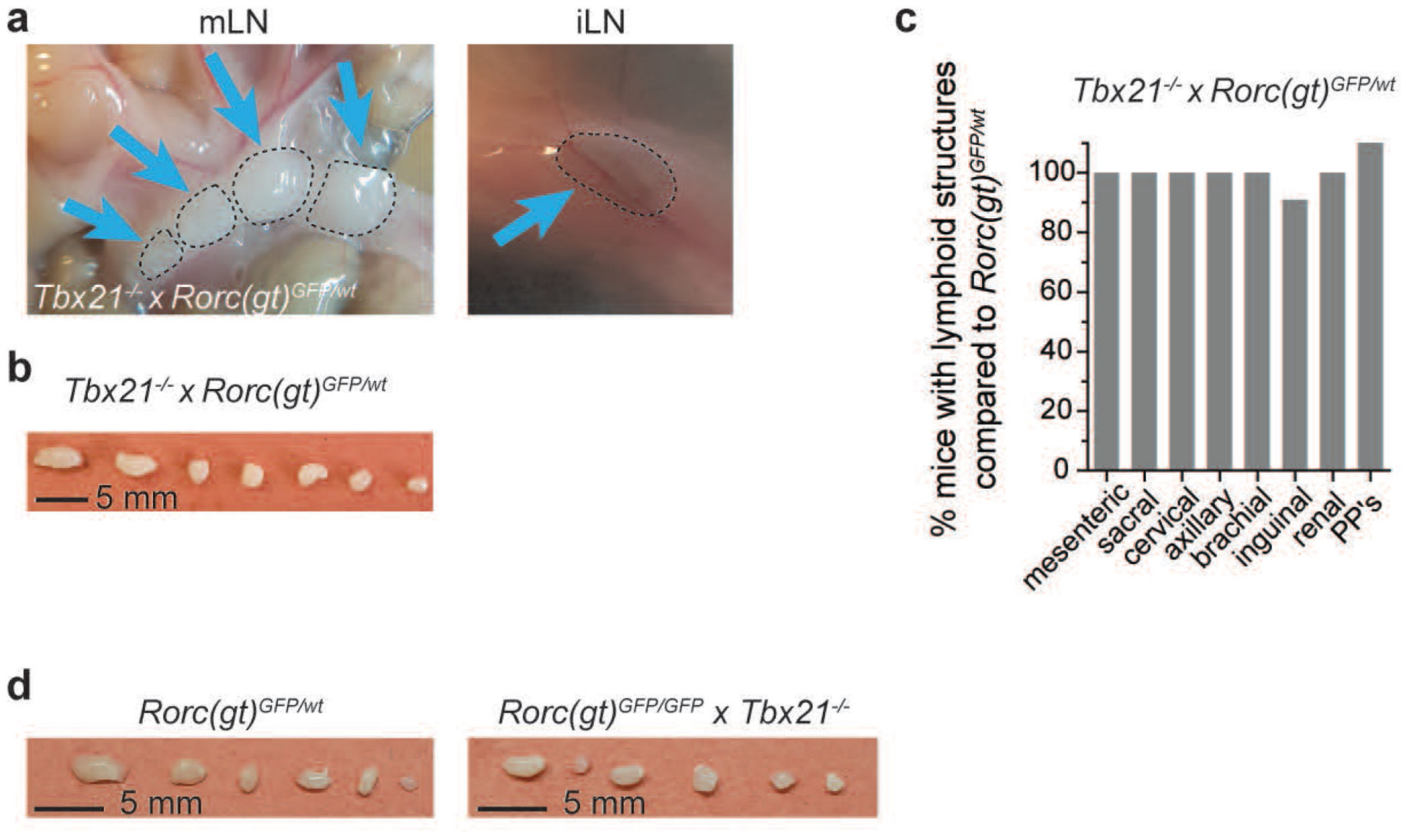
T-bet^-^deficient mice have normal LN development. **a**, *In vivo* photos of mesenteric or inguinal lymph nodes (mLN, iLN) of adult *Tbx21^-/-^ × Rorc(gt)^GFP/GFP^* mice or after isolation **(b)**. **(c)** Quantification (n=12) of frequencies of mice with lymphoid structures compared to control mouse strain in adult animals. PP’s, Peyer’s patches; nd, not detected. Data are representative of 2 independent experiments. **(d)** Photos of isolated mLN in indicated mouse strains. Data are representative of 2 independent experiments.

**Fig. 4:**
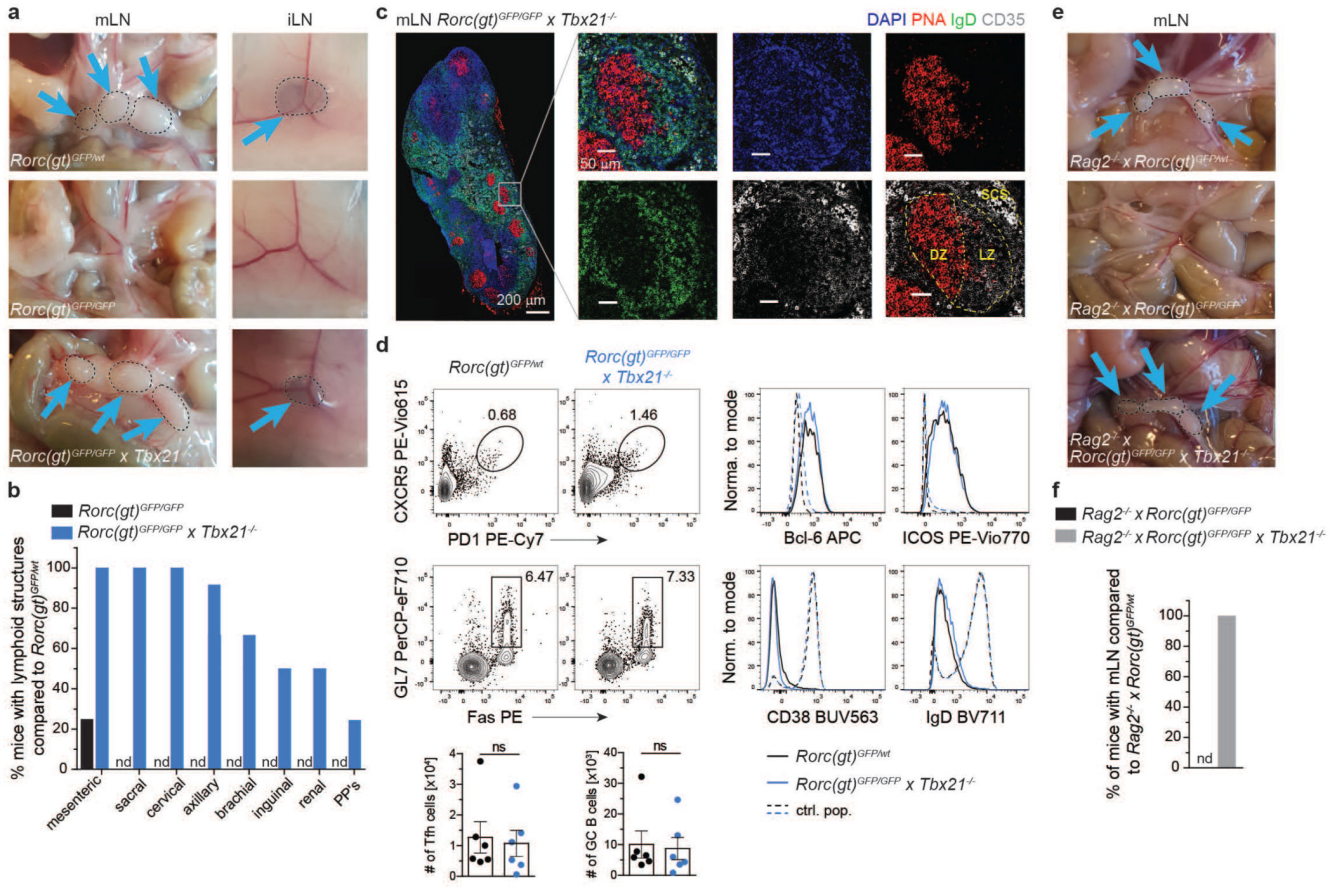
T-bet deficiency rescues formation of LN in RORγt-deficient mice. **a,** Photos of mesenteric or inguinal lymph nodes (mLN, iLN) of adult animals in indicated mouse strains. **(b)** Quantification (n=12) of frequencies of mice with lymphoid structures compared to control mouse strain *Rorc(gt)^GFP/wt^* in adult animals. PP’s, Peyer’s patches; nd, not detected. Data are representative of 2 independent experiments. (c) Immunofluorescence of mLN in *Rorc(gt)^GFP/GFP^* × *Tbx21^-/-^* mice. DZ, dark zone; LZ, light zone; SCS; subcapsular sinus. Data are representative of one experiment. (d) Representative flow cytometric plots of T follicular helper (Tfh) cells (upper row, gated as LD^-^CD45^+^CD3^+^CD4^+^CXCR5^+^PD1^+^) and germinal center (GC) B cells (lower row, gated as LD^-^CD45^+^CD3-B220^+^CD19^+^GL7^+^Fas^+^). Histograms show expression of Bcl-6 and ICOS in Tfh cells, control population consists of non-Tfh CD4^+^ T cells; and CD38 as well as IgD expression in GC B cells, control population consists of non-GC B cells. Black line represents *Rorc(gt)^GFP/wt^* mice, blue line represents *Rorc(gt)^GFP/GFP^ × Tbx21^-/-^* mice. Quantification of Tfh and GC B cells in the different mouse strains. Frequencies are shown as mean ± SEM, data are representative of 2 independent experiments, n=6. Mann-Whitney U test; ns, not significant. **(e)** Photos of mesenteric or inguinal lymph nodes (mLN, iLN) of adult animals in indicated mouse strains. **(f)** Quantification (n=12) of frequencies of mice with lymphoid structures compared to control mouse strain in adult animals; nd, not detected. Detailed statistics are available in source data.

Following up our finding of T-bet expression in fetal ILC3, we next investigated whether T-bet affected embryonic ILC3 functions, namely LN organogenesis, by analysing *Tbx21^-/-^ × Rorc(gt)^GFP/wt^* mice. *Rorc(gt)^GFP/wt^* reporter mice and *Rorc(gt)^GFP/GFP^* mice (lacking RORγt) were used as control. Whereas RORγt deficiency in *Rorc(gt)^GFP/GFP^* mice led to impairment of peripheral LN formation (Fig. 4, a and b), as previously reported^12,13^, LN generation in adult *Tbx21^-/-^ × Rorc(gt)^GFP/wt^* mice was largely normal (Extended Data fig. 4, a - c)^16^. It is well established that T-bet and RORγt are co-expressed within adult ILC3 where they cross-regulate each other^15,16,20^. in order to investigate the relevance of both TFs for fetal ILC3 development, we generated double knockout mice. Surprisingly, analysis of *Rorc(gt)^GFP/GFP^ × Tbx21^-/-^* mice, lacking both T-bet and RORγt, resulted in complete rescue of sacral, cervical, axillary and mesenteric LN, whereas emergence of brachial, inguinal and renal LN occurred in around 30-60% and Peyer’s patches (PP) in 20% of the animals (Fig. 4, a and b, and Extended Data fig. 4d). mLN from *Rorc(gt)^GFP/GFP^ × Tbx21^-/-^* mice displayed normal LN architecture and functions, as demonstrated by the presence of germinal centres (GCs), marked by PNA-binding B cell clusters surrounded by IgD^+^ naive B cells. GCs displayed a discrete segregation of dark and light zone, as shown by staining follicular dendritic cells in the latter one with CD35 (Fig. 4c). Flow cytometric analysis of mLN from *Rorc(gt)^GFP/GFP^ × Tbx21^-/-^* mice further identified similar numbers of *CXCR5*^+^PD1^+^ T follicular helper (Tfh) cells expressing ICOS and Bcl-6 as well as IgD^-/lo^CD38^-^Fas^+^GL7^+^ GC B cells, as compared to control mice, pinpointing functional competence of mLN in these mice (Fig. 4d). In order to rule out a possible role for T or B cells in driving LN development in the absence of T-bet and RORγt, we analysed *Rorc(gt)^GFP/GFP^ × Tbx21^-/-^ × Rag2^-/-^* mice and observed mLN development in all mice analysed (Fig. 4, e and f). in sum, our data show that in the presence of RORγt, T-bet does not influence embryonic LTi cell functions. in the absence of RORγt however, T-bet inhibits LN formation in a T cell- and B cell-independent manner as evidenced by rescue of LN in mice double deficient for RORγt and T-bet.

### ILCP accumulate due to impaired ILC1/3 differentiation

**Extended Data Fig. 5:**
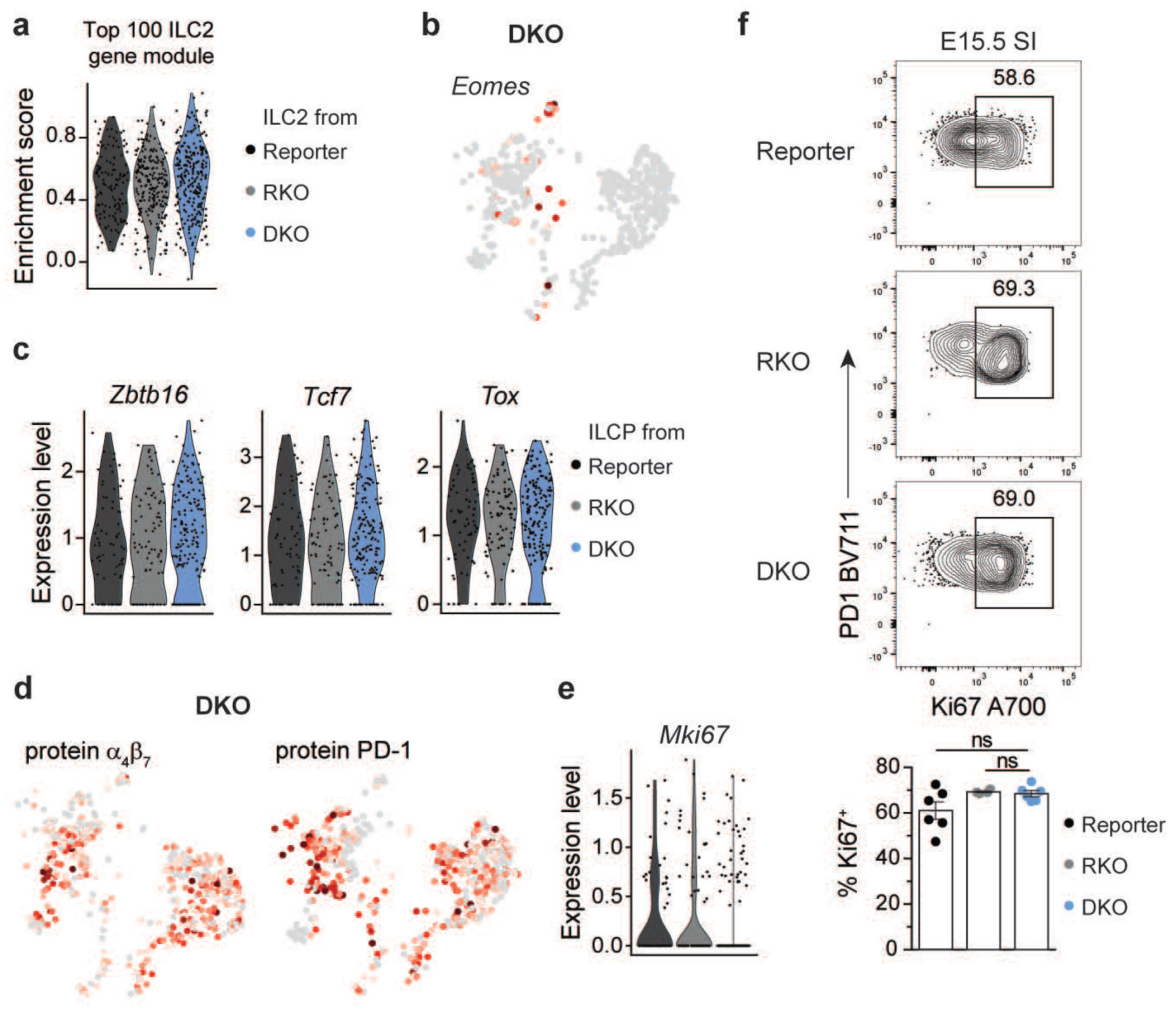
Comparative gene expression of ILCP from reporter, RKO and DKO mice. **a**, Violin plots for enrichment score of gene modules of top 100 differentially expressed genes from reporter ILC2 within ILC2 cluster in indicated mouse strains. Statistical significance was calculated using two-sided Wilcoxon test with Bonferroni correction, all ns. **(b)** *Eomes* expression in DKO mice projected on UMAP. **(c)** Expression of selected ILCP-associated genes in cells from ILCP cluster in indicated mouse strains. **(d)** Expression of selected proteins on UMAP projection analysed by CITE-Seq. **(e)** Violin plot for *Mki67* transcripts among ILCP in all mouse strains, legend see in **(c)**. **(f)** Representative flow cytometry of Ki67 expression in LinLD^-^ CD45^+^CD127^+^ROR□t^-^PD1^hi^PLZF^hi^ cells from E15.5 SI (reporter n=6, RKO n=4, DKO n=6 from 2 independent experiments). Quantification as mean ± SEM with Kruskal-Wallis significance and Dunn’s correction. Detailed statistics are available in source data.

**Fig. 5:**
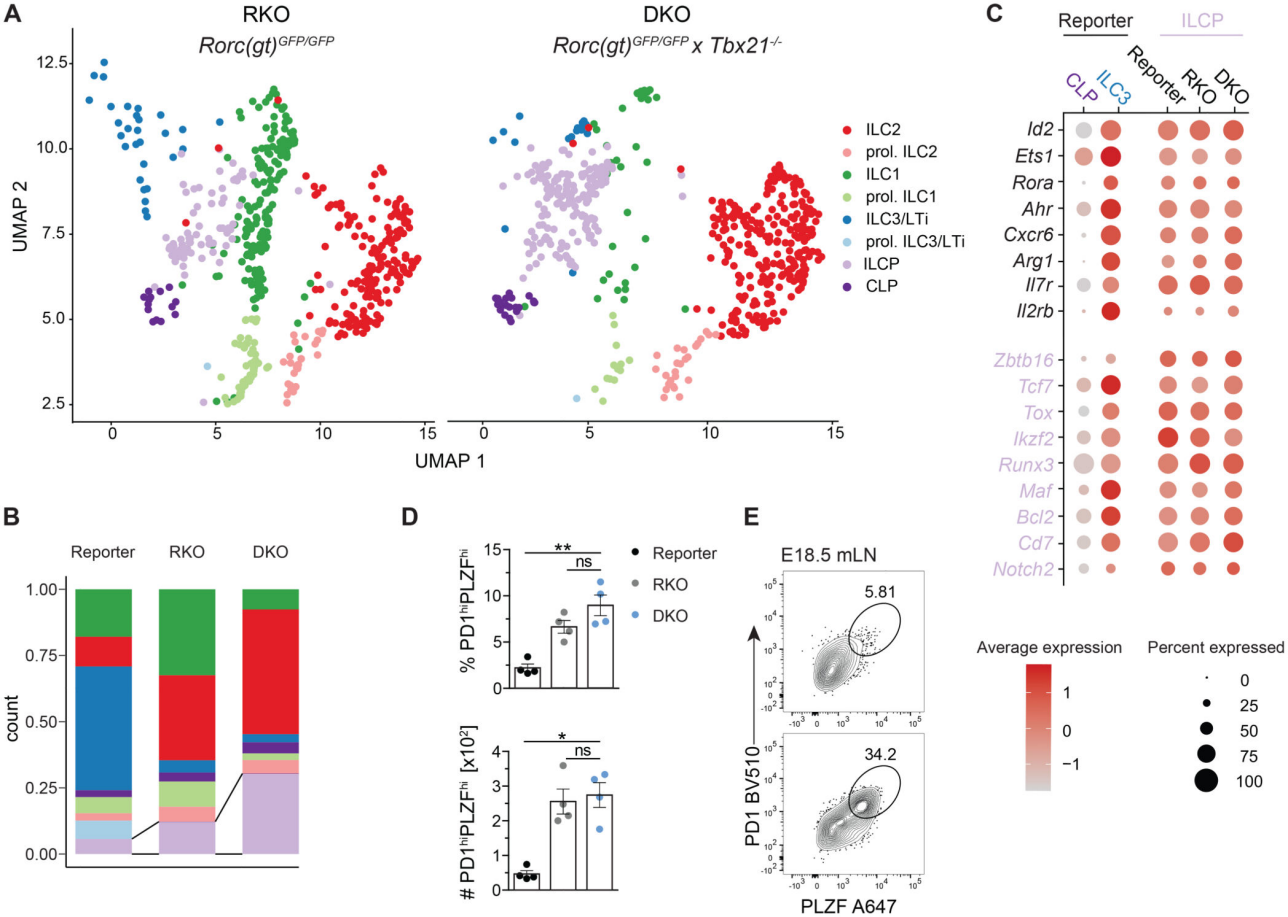
Single-cell sequencing of fetal ILC populations reveals accumulation of ILCP in *Rorc(gt)^GFP/GFP^ × Tbx2^-/-^* mice. Viable Lin(CD19, CD3, CD5, F4/80, FceRIa, Gr-1)^-^ CD45^+^ cells expressing IL-7 receptor (CD127) and/or the IL-2 receptor subunit beta (CD122) isolated from the small intestine (SI) of E18.5 *Rorc(gt)^GFP/GFP^* (RKO) and *Rorc(gt)^GFP/GFP^ × Tbx21^-/-^* (DKO) embryos were sort-purified by flow cytometry, and a single-cell expression library was generated using 10x Genomics. **a**, UMAP dimensional reduction projection identifies eight distinct clusters. **(b)** Quantification of single cells mapping to correspondent clusters (legend see in **(a)** in indicated mouse strains. **(c)** Selected gene expression within clusters and mouse strains. CLP and ILC3 from reporter mice compared to ILCPs of all strains. Color scale represents average expression, dot size visualizes fraction of cells within the cluster expressing the gene. **(d)** Quantification of PD1 ^hi^PLZF^hi^ ILCP among LinLD^-^CD45^+^CD127^+^ and/or CD122^+^ as mean ± SEM with Kruskal-Wallis significance and Dunn’s correction in the respective mouse strains isolated from E18.5 small intestine. Data are representative of 2 independent experiments (n=4). P values are provided in source data. **(e)** Representative flow cytometric plot of pooled E18.5 mLN anlagen gated on LinLD^-^CD45^+^CD127^+^ and/or CD122^+^ cells. Detailed statistics are available in source data.

To investigate whether LN organogenesis in *Rorc(gt)^GFP/GFP^ × Tbx21^-/-^* mice was associated with the existence of an ILC population that retained LTi cell functions, we performed single-cell RNA sequencing on LinLD^-^CD45^+^CD122^+^ and/or CD127^+^ ILCs Isolated from E18.5 SI of *Rorc(gt)^GFP/GFP^ × Tbx21^-/-^* (DKO), as compared to *Rorc(gt)^GFP/GFP^* (RKO) or *Rorc(gt)^GFP/wt^* (reporter) mice. Combined analysis of equal numbers of cells from the three mouse strains revealed the presence of overlapping ILC clusters with comparable gene expression signatures in RKO and DKO as in reporter mice, although with clearly different distributions (Fig. 1a and 5a–b). As expected, ILC3 populations were drastically diminished in both RKO and DKO mice due to RORγt deficiency. Lack of ILC3 was paralleled by a conspicuous increase of ILC2 in both RKO and DKO strains and of ILC1/NK selectively in RKO, highlighting competitive cross-regulation between the different ILC lineages (Fig. 5, a and b). Besides increased frequencies, ILC2 transcriptional signatures were minimally affected in RKO and DKO, as compared to reporter mice (Extended Data fig. 5a). Because of T-bet deficiency, DKO mice were defective of the ILC1 cluster, while few cells with *Eomes* transcripts could be still detected (Extended Data fig. 5b). When analysing the progenitor populations, we observed a strong overrepresentation of the ILCP cluster in DKO mice, with equal frequency of the CLP (Fig. 5, a and b). ILCP derived from all strains displayed comparable expression of ILC-lineage markers like *Id2*, *Ets1*, *Rora*, *Ahr*, *CXCR6*, *Arg1*, *Il7r* and *Il2rb*, as well as of ILCP-associated genes *Zbtb16*, *Tcf7* and *Tox* (Fig. 5c and Extended Data fig. 5c). Additionally, α_4_β_7_ and PD-1 could be detected on the surface of DKO ILCP (Extended Data fig. 5d), as also seen in reporter mice. Accordingly, PLZF^hi^ PD1^hi^ ILCP were detected by flow cytometry among LinLD^-^CD127^+^ and/or CD122^+^ cells derived from E18.5 SI and mLN anlagen and their frequency and absolute number were increased in DKO and RKO, as compared to reporter mice (Fig. 5d and e). ILCP accumulation in DKO mice was not associated to increased proliferation, as Ki67 expression was comparable in ILCP from the three strains (Extended Data fig. 5e and f).

These data suggest that under unperturbed conditions, there is continuous ILCP differentiation towards the mature ILC lineages, as shown in reporter mice; on the other hand, in the absence of RORγt and T-bet, ILCP cannot further differentiate towards ILC1 or ILC3 lineages and therefore accumulate at this differentiation stage.

### ILCP of DKO mice are enriched in cells with LTi signatures

**Extended Data Fig. 6:**
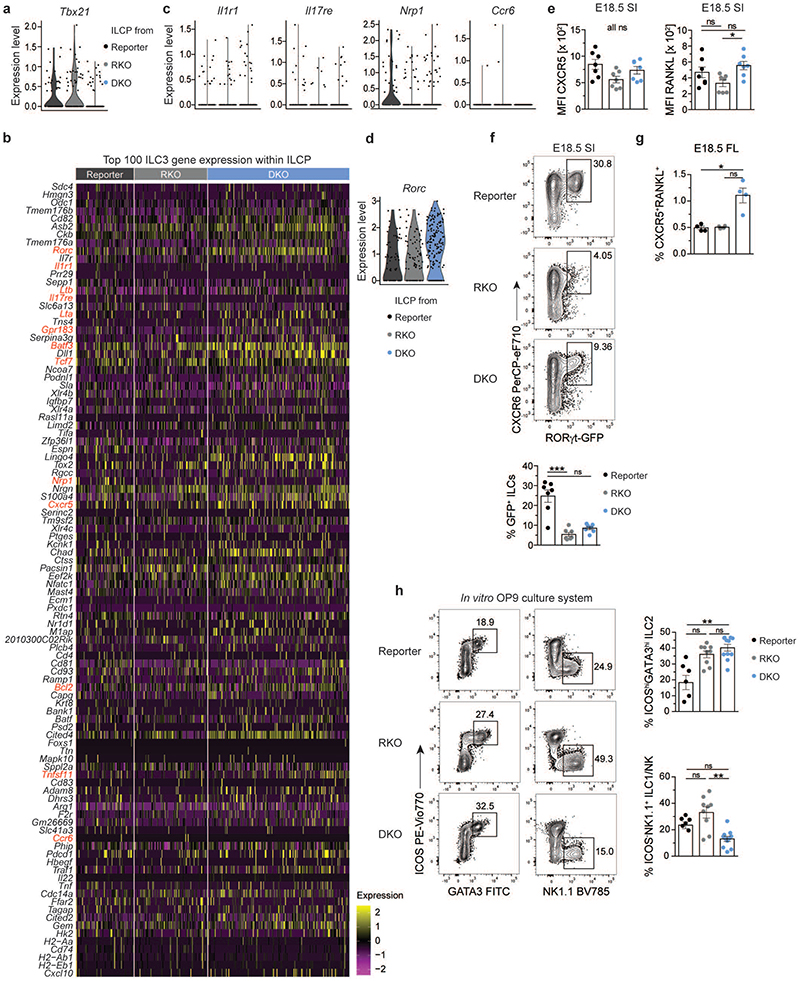
Gene and protein expression patterns in ILCP of reporter, RKO and DKO mouse strains. **a**, Expression levels of *Tbx21* within ILCP of all strains. **(b)** Gene expression profiles of the top 100 genes differentially expressed in ILC3 of reporter mice within cells from ILCP cluster in the different mouse strains. **(c)** Violin plots depicting expression of selected transcripts in ILCP of the three mouse strains, legend see in (A). **(d)** Violin plot depicting *Rorc* expression within ILCP of indicated mouse strains. **(e)** Geometric mean fluorescence intensity of *CXCR5* and RANKL within LinLD^-^CD45^+^CD127^+^ and/or CD122^+^ cells isolated from E18.5 SI (n=7 from 2 independent experiments) depicted as mean ± SEM and Kruskal-Wallis significance with Dunn’s correction. Data from two independent experiments. **(f)** Representative flow cytometry from E18.5 SI of LinLD^-^CD45^+^ CD127^+^ and/or CD122^+^ cells and quantification as mean ± SEM of GFP^+^ cells (n=7 from 2 independent experiments) with Kruskal-Wallis significance and Dunn’s correction. **(g)** Frequencies of *CXCR5*^+^RANKL^+^ cells among LinLD^-^CD45^+^CD127^+^ and/or CD122^+^ cells in fetal liver (FL) of E18.5 embryos (n=4 from 2 independent experiments) with Kruskal-Wallis significance and Dunn’s correction. ^(h)^ *in vitro* differentiation of E14.5 fetal liver-derived ILC progenitors on OP9 stromal cells for 5-8 days in the presence of SCF and IL-7 and analysis by flow cytometry. Quantification of LinLD^-^CD45^+^ICOShiGATA3hi ILC2 and LinLD^-^CD45^+^ICOS-NK1.1^+^ group 1 ILCs cells in the different mouse strains shown as mean ± SEM. Data are representative of 2-3 independent experiments (reporter n=5 from 2 independent experiments, RKO n=9 and DKO n=11 from 3 independent experiments). Kruskal-Wallis significance and Dunn’s correction. Detailed statistics are available in source data.

**Fig. 6:**
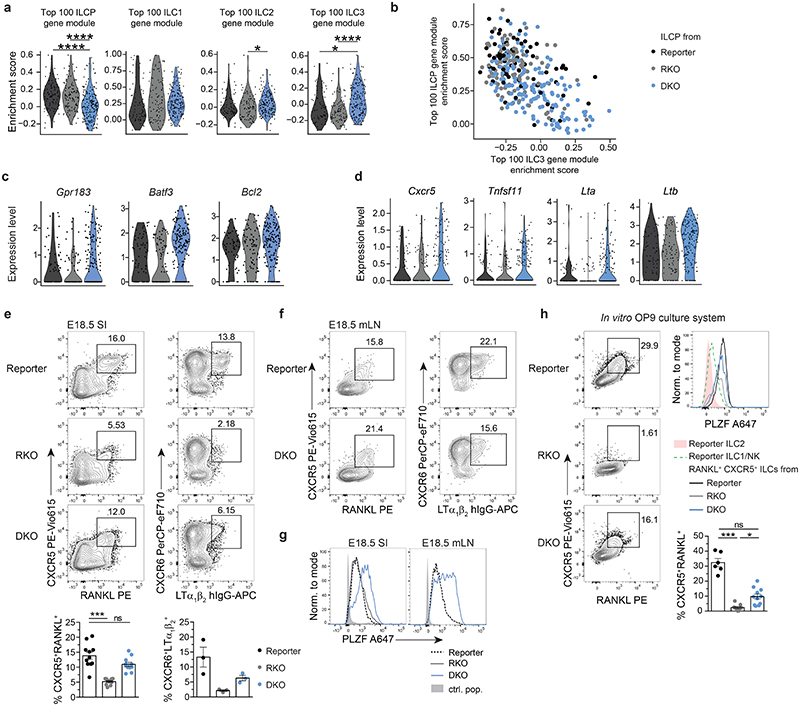
PLZF^hi^ ILCP from DKO mice are enriched in cells with LTi signatures. **a**, Violin plots for enrichment score of gene modules of top 100 differentially expressed genes from ILCP or ILC1, ILC2, ILC3 cluster, defined in reporter mice, in single cells from ILCP cluster in indicated mouse strains. E18.5 scRNA-seq dataset, legend see in **(b)**. Statistical significance was calculated using two-sided Wilcoxon test with Bonferroni correction. **(b)** Co-enrichment plots of ILCP versus ILC3 gene modules within single cells of ILCP cluster in mouse strains. **(c,d)** Expression of selected ILC3-associated genes in single cells of ILCP cluster. See legend in B. **(e)** Flow cytometric analysis from E18.5 SI after gating on LinLD^-^CD45^+^CD127^+^ and/or CD122^+^ cells. Quantification of *CXCR5*^+^RANKL^+^ (n=11, 3 independent experiments) and CXCR6^+^LTα_1_β_2_^+^ (n=3, one experiment) cells in the different mouse strains shown as mean ± SEM, Kruskal-Wallis significance and Dunn’s correction. **(f)** Representative flow cytometric plot of pooled E18.5 mLN anlagen gated on LinLD^-^CD45^+^CD127^+^ and/or CD122^+^ cells. **(g)** Representative flow cytometric histogram of LinLD^-^CD45^+^CD127^+^ and/or CD122^+^CXCR6^+^ cells; control population (ctrl. pop.) defined as CD127^-^CD122^-^ of reporter mice. **(h)** In vitro differentiation of E14.5 fetal liver-derived ILC progenitors on OP9 stromal cells after culture for 5-8 days in the presence of SCF and IL-7 and analysis by flow cytometry. Representative flow cytometry plots of *CXCR5*^+^RANKL^+^ cells among LinLD^-^CD45^+^GATA3^hi-neg^NK1.1^-^ in the different mouse strains. Quantification shown as mean ± SEM, data are representative of 2-3 independent experiments (reporter n=5, 2 independent experiments, RKO n=9 and DKO n=11, 3 independent experiments). Kruskal-Wallis significance and Dunn’s correction. Detailed statistics are available in source data.

We next examined how deficiency of RORγt and T-bet affects ILCP transcriptional profile and differentiation commitment by analysing the enrichment score of DKO, RKO and reporter ILCP for ILCP, ILC1, ILC2 or ILC3 gene modules (Fig. 6a). We found that RKO ILCP were enriched in ILC1 and ILC2, but not in ILC3 module (Fig. 6a), as expected from RORγt deficiency. Accordingly, ILCP cells expressing *Tbx21* transcripts were more abundant in RKO than of reporter mice (Extended Data fig. 6a), suggesting that in the absence of RORγt counterbalance, ILCP maturation is skewed towards ILC1 lineage. Conversely, in DKO mice additional lack of T-bet resulted in an increased frequency of ILCP with a higher enrichment score for ILC3 (Fig. 6b). Interestingly, DKO ILCP with high enrichment for ILC3 genes exhibited low enrichment score for the ILCP gene module (Fig. 6a and 6b), suggesting that lack of T-bet facilitates progression of ILCP towards the ILC3 lineage. We then analysed which among the top 100 genes defining the ILC3 core module were upregulated in ILCP from DKO mice (Extended Data fig. 6b). DKO ILCP displayed no or only little expression of *Il1r1*, *Il17re*, *Nrp1* or *Ccr6*, similar to their ILCP counterparts from reporter or RKO strains (Extended Data fig. 6b and c). In contrast, besides *Gpr183* and *Batf3* and *Bcl2* (Fig. 6c), an increased fraction of DKO ILCP expressed transcripts for genes associated with LTi functions, namely *Lta*, *Ltb*, *Cxcr5* and *Tnfsf11* (encoding RANKL) (Fig. 6d). Of note, although lack of ILC3 advocated for a loss of RORγt-driven functions in both RKO and DKO mice (Fig. 5, a and b), residual *Rorc* transcripts could still be observed within the ILCP from RKO and DKO strains (Extended Data fig. 6b and d).

To validate the data obtained by scRNA-seq, we performed flow cytometry of LinLD^-^ CD127^+^ and/or CD122^+^ cells derived from E18.5 SI and mLN anlagen. A population of CXCR6^+^ cells expressing surface LTi functional molecules, namely RANKL, *CXCR5* and the heterotrimer LTα_1_β_2_, was present in the SI and mLN of DKO mice at comparable higher frequencies than in reporter mice where CXCR6^+^ RANKL^+^ CXCR5^+^ LTα_1_β_2_^+^ cells largely comprised of ILC3 (Fig. 6e and f). Besides significant differences in frequency, the MFI of RANKL or *CXCR5* was slightly increased in DKO versus RKO ILCP (Extended Data fig. 6e). In line with detection of *Rorc* transcripts by scRNA-seq, a consistent GFP signal indicating Rorc promoter activity was found in CXCR6^+^ cells from DKO and at lower frequencies in RKO mice (Extended Data fig. 6f). In support of the scRNA-seq data, DKO CXCR6^+^ cells expressed high levels of PLZF, confirming their ILCP identity (Fig. 6g). Thus, both scRNA-seq and flow cytometric analysis identified an ILCP population with LTi signatures in SI and mLN anlage of DKO mice. The frequency of *CXCR5*^+^ RANKL^+^ ILCs derived from FL of DKO was also higher as compared to RKO or reporter mice, although generally lower than in SI and mLN anlage (Fig. 6e and Extended Data fig. 6g). This shows that skewed ILCP differentiation towards acquisition of LTi signatures in DKO mice seems to occur already in the FL (Extended Data fig. 6g). To validate this concept, we performed *in vitro* differentiation assays culturing FL progenitors derived from the three strains on OP9 feeder cells in the presence of SCF and IL-7. After one week culture, all ILC lineages could be generated *in vitro* using progenitors from reporter mice (Fig. 6h and Extended Data fig. 6h). in line with the data observed *ex vivo*, ILC2 differentiation was enhanced in cultures from RKO and DKO mice, while generation of NK1.1 ^+^ cells (marking both ILC1 and NK cells) was reduced in DKO as compared to RKO mice (Extended Data fig. 6h). Importantly, cultures from DKO, but not from RKO, progenitors enabled the generation of a population of RANKL^+^CXCR5^+^ cells, which co-expressed PLZF (Fig. 6h).

Altogether, these data imply that lack of RORγt prevents the development of fully mature ILC3; however, in the absence of T-bet, it still enables the differentiation of ILCP with selected LTi features.

### PLZF^hi^ CXCR6^+^ GFP^+^ cells persist in adult DKO mice

**Extended Data Fig. 7:**
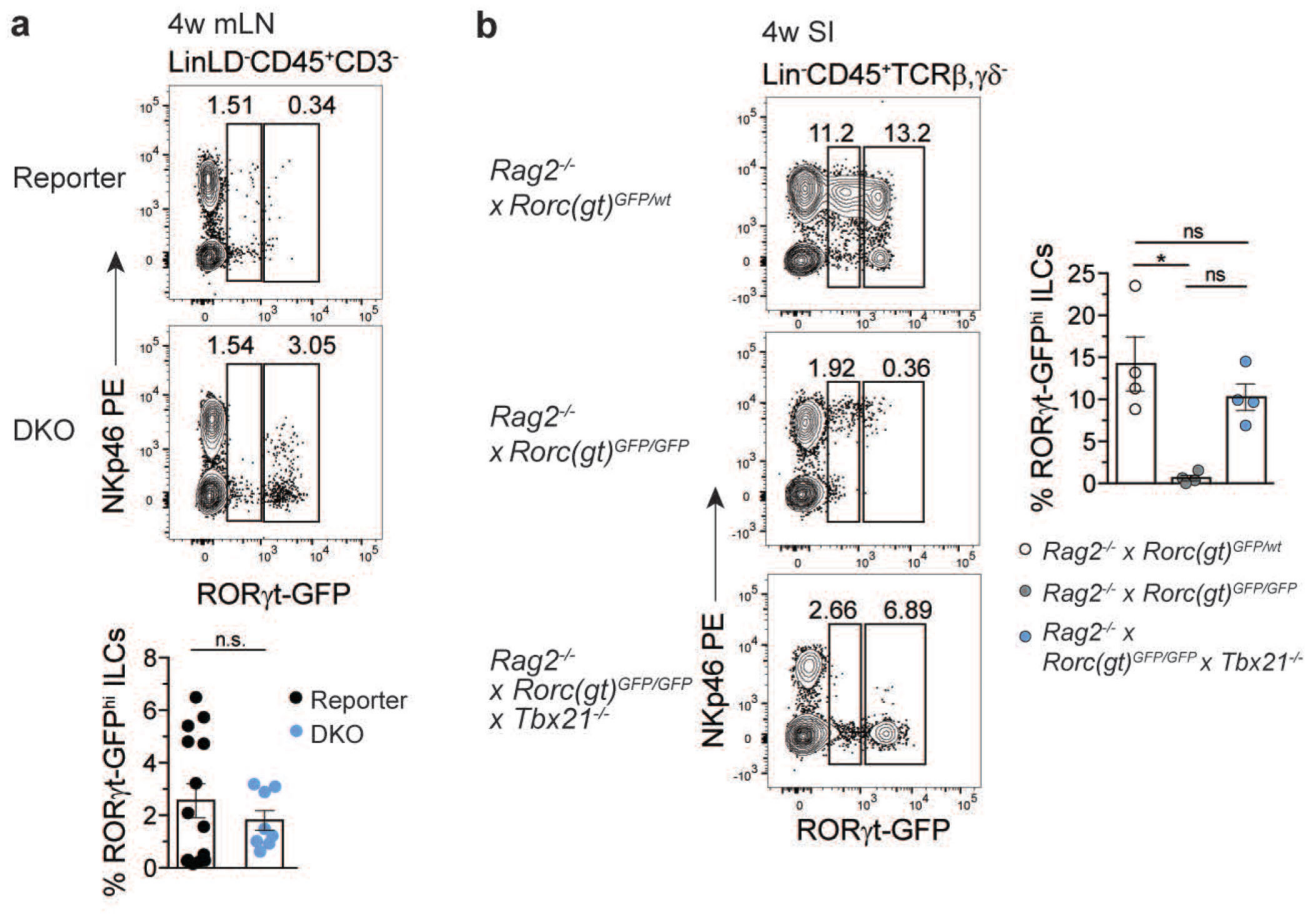
GFP^+^ ILCs persist in double knockout mouse models and develop independently from RAG proteins. **a**, Flow cytometry representative plots of mLN from 4-week-old mice of indicated mouse strains. Quantification of frequencies from two independent experiments depicted as mean ± SEM (reporter n=14, DKO n=8 from 4 independent experiments). Kruskal-Wallis testing with Dunn’s multiple comparison correction. **(b)** Representative flow cytometry of cells from SI isolated from 4-week-old mice. Quantification of frequencies from 2 independent experiments as mean ± SEM, Kruskal-Wallis testing with Dunn’s multiple correction, n=4. *P* values are provided in source data. Detailed statistics are available in source data.

**Fig. 7:**
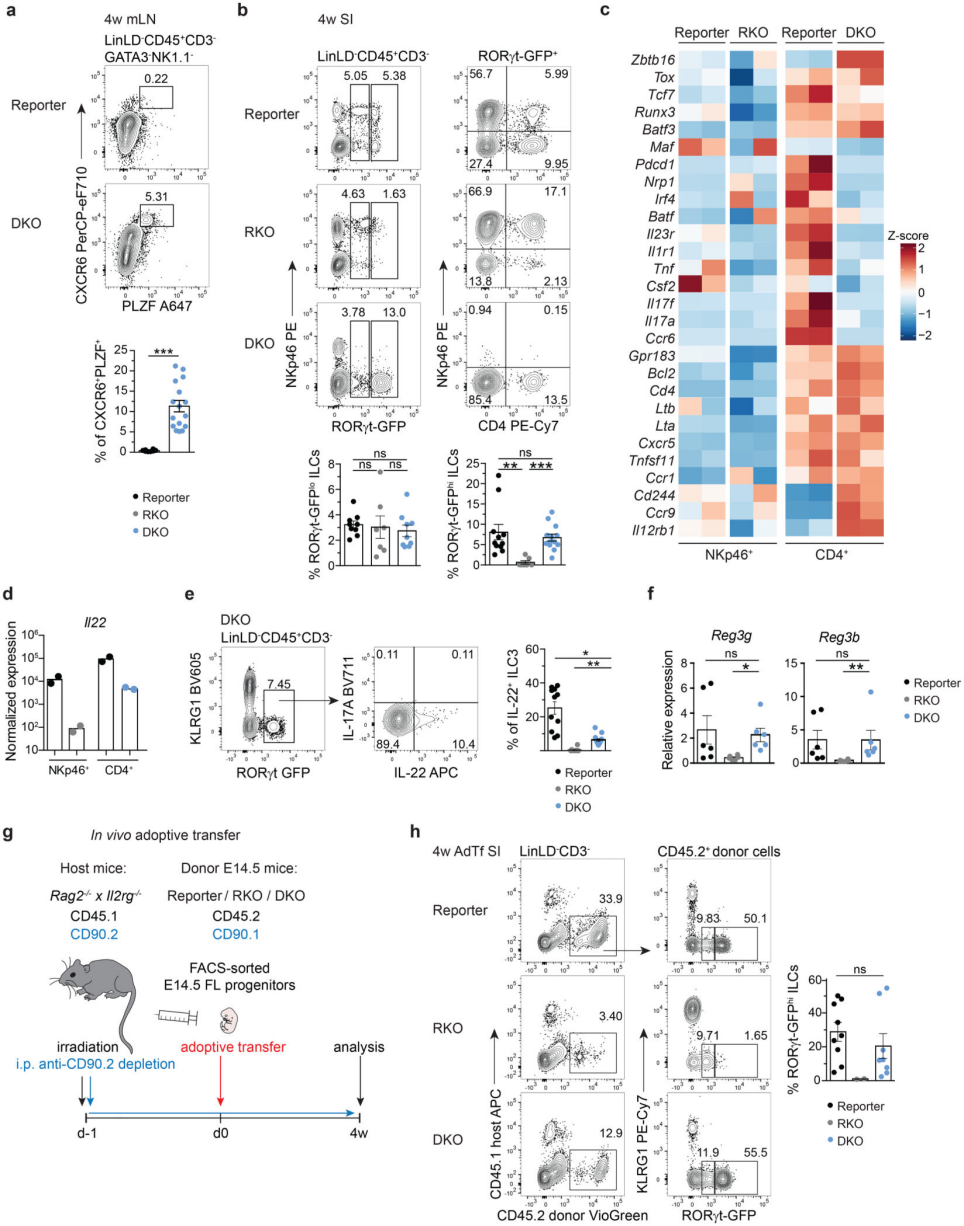
PLZF^hi^ CXCR6^+^ GFP^+^ cells persist in mLN and intestine of adult *Rorc(gt)^GFP/GFP^* × Tbx21^-/-^ mice. **a**, Flow cytometry representative plots of mLN from 4-week-old reporter (*Rorc(gt)^GFP/wt^*) and DKO (*Rorc(gt)^GFP/GFP^ × Tbx21^-/-^*) mice. Quantification of frequencies from 3 independent experiments (reporter n=8, DKO n=16), mean ± SEM and Mann-Whitney-U testing. **(b)** Representative flow cytometry plots of SI from indicated mouse strains at 4 weeks of age. Quantification of RORγt-GFP^lo^ and RORγt-GFP^hi^ populations depicted as mean ± SEM, Kruskal-Wallis testing with Dunn’s multiple correction, reporter n=9, RKO n=7, DKO n=9 from 3 independent experiments. See legend in **(a)**. **(c)** Heatmap of bulk RNA-seq of RORγt-GFP^+^ population sort-purified according to NKp46 or CD4 expression isolated from SI of 4-week-old mice. Selected gene transcripts are shown as Z-score. (d) Normalized expression values of *Il22* transcripts from bulk RNA-seq dataset and **(e)** IL-22 protein expression in SI of DKO mice determined by flow cytometry. Quantification of frequencies from 3 independent experiments (reporter n=11, RKO n=14, DKO n=15), mean ± SEM and Kruskal-Wallis testing with Dunn’s multiple correction. **(f)** Expression of *Reg3g* and *Reg3b* in intestinal epithelial compartment determined by quantitative PCR. Values are normalized to housekeeping genes *Actb*, *Hprt* and *Gapdh*. Each symbol represents an individual mouse. Data show mean ± SEM, Kruskal-Wallis testing with Dunn’s multiple correction, n=6. **(g)** Schematic overview of experimental setup for adoptive transfer experiment. FL, fetal liver. **(h)** Representative flow cytometry in SI of *Rag2^-/-^ × II2rg^-/-^* mice 4 weeks after adoptive transfer and quantification of frequencies of RORγt-GFP^hi^ ILCs as mean ± SEM (reporter n=9, RKO n=2, DKO n=8), Kruskal-Wallis significance with Dunn’s correction. Data are representative of 2 experiments. Detailed statistics are available in source data.

The identification of ILCP with LTi features in embryos of RORγt and T-bet double deficient mice prompted us to investigate whether such a population would persist into adulthood. PLZF^hi^ CXCR6^+^ cells were present in the mLN of 4 week-old DKO (Fig. 7a). In line with the data of the E18.5 SI, a GFP signal could be detected in mLN and SI of adult DKO, as well as in SI of adult RKO mice (Fig. 7b and Extended Data fig. 7a). Interestingly, in contrast to GFP^lo^ cells (present in both RKO and DKO mice), *GFP^hi^* ILCs were absent in the SI of RKO, but detected in similar frequencies in DKO and reporter mice and partially expressed CD4, but not NKp46 (Fig. 7b). Likewise, *GFP^hi^* ILCs appeared in the SI of *Rag2^-/-^* DKO but not in *Rag2^-/-^* RKO mice (Extended Data fig. 7b). Bulk RNA-sequencing of NKp46^+^ and of CD4^+^ GFP^+^ populations sorted from SI of adult reporter, RKO and DKO mice underlined the observations gained in the E18.5 single-cell approach. DKO CD4^+^ GFP^+^ cells displayed high *Zbtb16*, *Tox* and *Tcf7* expression, while lacking other ILC3-related genes including *Il1r1* and *Ccr6*. Conversely, CD4^+^ GFP^+^ cells from DKO and reporter mice still displayed comparable levels of transcripts for central LTi cell molecules, such as *Lta*, *Ltb*, *Tnfsf11* and *Cxcr5* (Fig. 7c). Importantly, *Il22*, but not Il17a, transcripts were restored to similar amounts in DKO CD4^+^ GFP^+^ cells as in NKp46^+^ ILC3 from reporter mice (Fig. 7c and e). Along this line, IL-22, but not IL-17A, protein was detected in DKO CD4^+^ GFP^+^ cells and was sufficient to rescue expression of *Reg3g* and *Reg3b* encoding antimicrobial peptides in the intestinal epithelium (Fig. 7f).

These data show that in the absence of T-bet and RORγt, PLZF^hi^ ILCP with LTi signatures and residual *Rorc* promoter activity develop during embryonic life and persist into adulthood. Based on these data, we next asked whether transfer of DKO fetal progenitors could give rise to such a population in adult mice. To this end, we adoptively transferred FL progenitors from reporter, RKO or DKO CD90.1^+^ CD45.2^+^ mice into irradiated CD90.2^+^ CD45.1^+^ *Rag2^-/-^Il2rg^-/-^* hosts. Irradiated recipient mice were treated bi-weekly with anti-CD90.2 antibody in order to deplete residual radio-resistant host ILCs^44–46^ and analysed 4 weeks after reconstitution (Fig. 7g). In line with the phenotype observed *ex vivo* in adult DKO mice, only DKO progenitors efficiently reconstituted a population of GFP^hi^ cells at similar frequency to the one observed in reporter mice (Fig. 7h).

Collectively, we have shown that deficiency of T-bet and RORγt enables the generation and accumulation of embryonic PLZF^hi^ ILCP, which can persist into adulthood. Lack of T-bet facilitates ILCP expression of molecules conferring LTi function and IL-22, thereby rescuing LN formation during embryogenesis and promoting the production of antimicrobial peptides in the intestinal epithelium postnatally.

### RORα promotes LN development in DKO mice

**Extended Data Fig. 8:**
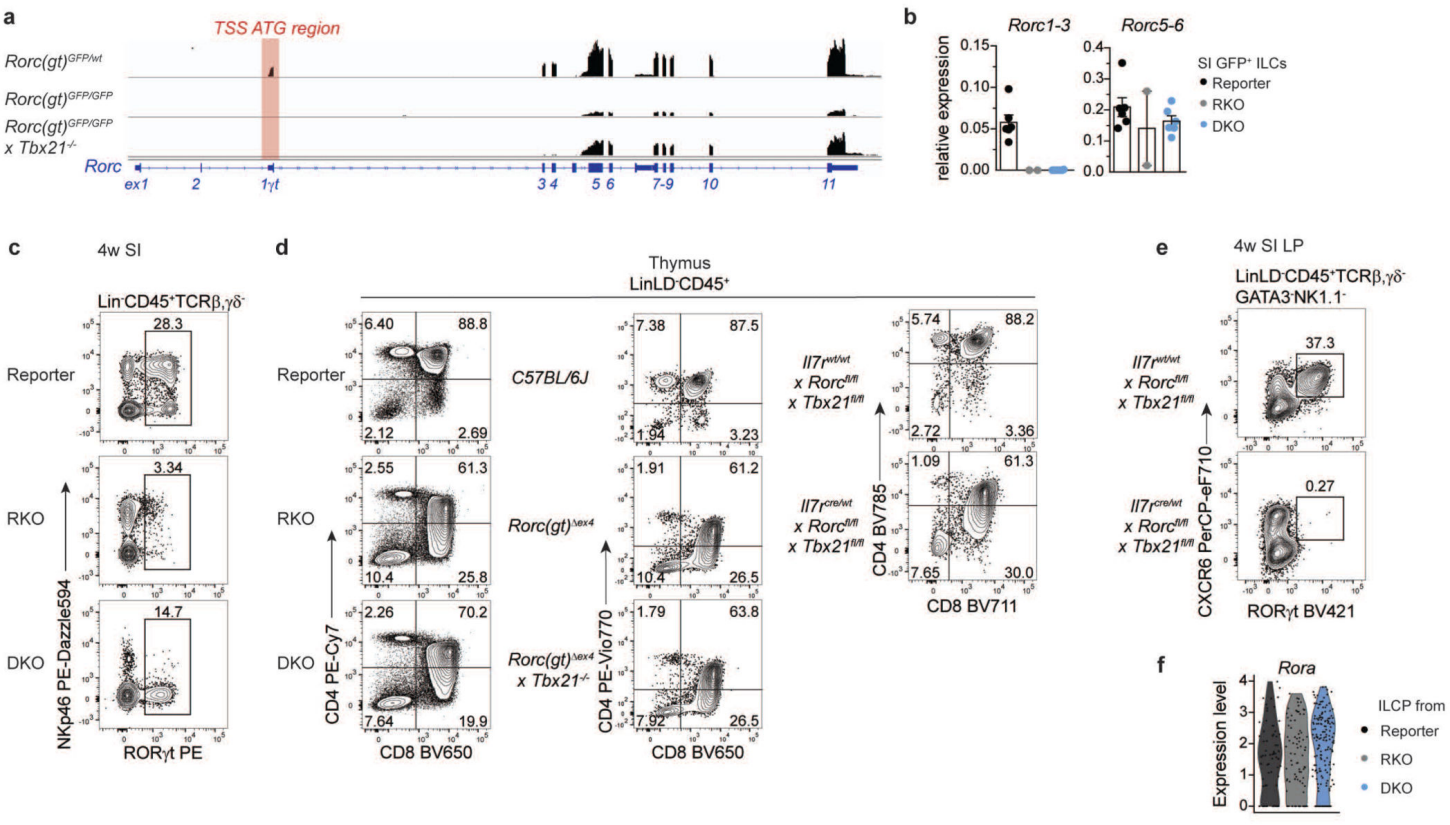
Thymic CD4^+^CD8^+^ compartments are not restored in RORγt/T-bet double-deficient mice. **a**, Mapping of reads detected by bulk RNA-seq to the mm10 mouse *Rorc* locus in indicated mouse strains. Red box designates localization of transcriptional start site (TSS) ATG of exon 1**γ**t. **(b)** Expression of *Rorc* exon1-3 and *Rorc* exon5-6 junctions in small intestinal LinLD^-^ CD45^+^CD3-GFP^+^ ILCs of 4-week-old mice determined by quantitative PCR. Values are normalized to housekeeping gene *Gapdh*. Each symbol represents an individual mouse. Data show mean ± SEM, reporter and DKO n=6, RKO n=2 examined over 2 independent experiments. **(c,d,e)** Representative flow cytometry plots of indicated populations and compartments isolated from 4-week-old mice. **(f)** Violin plot of *Rora* expression within ILCP of designated mouse strains from E18.5 scRNA-seq dataset. Detailed statistics are available in source data.

**Fig. 8:**
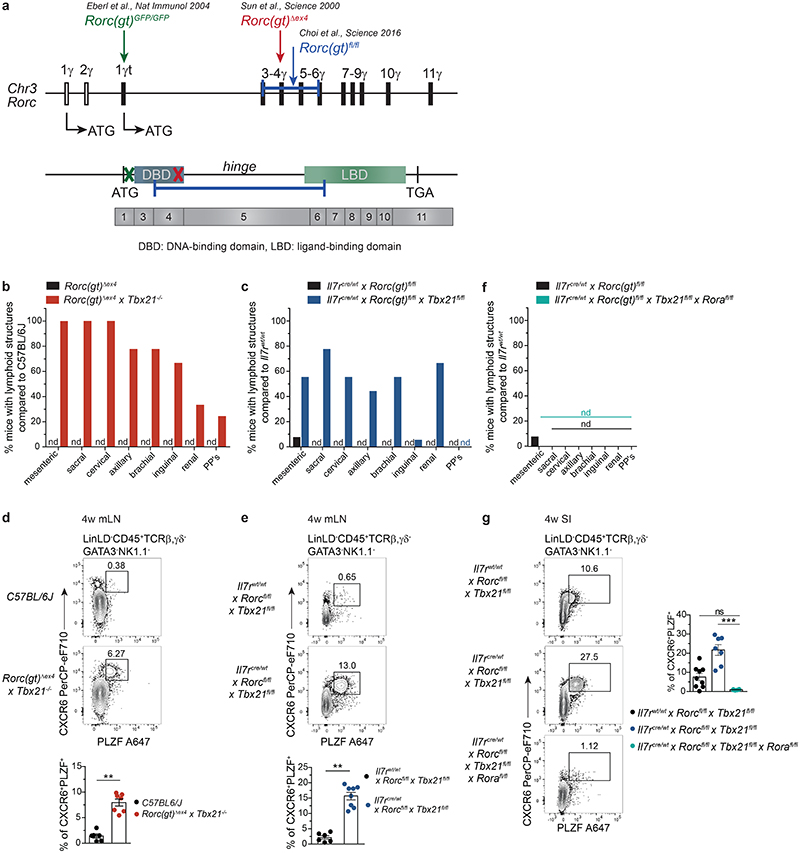
LN formation and accumulation of PLZF^+^ ILCP is independent of DNA- and ligand-binding domains of RORγt and promoted by RORα in the absence of T-bet. **a**, Schematic overview on *Rorc* locus on chromosome 3 (*Chr3*) and genetic targets in designated mouse strains. **(b,c,f)** Quantification (n=12) of frequencies of mice with lymphoid structures compared to control mouse strain in adult animals. PP’s, Peyer’s patches; nd, not detected. **(d,e,g)** Flow cytometry representative plots of mLN **(d,e)** or SI **(g)** from 4-week-old mice of indicated mouse strains. **(d)** Quantification of frequencies from 2 independent experiments as mean ± SEM, C57BL/6J n=6, *Rorc(gt)^Δex4^ × Tbx21^-/-^* n=7, Mann-Whitney-U testing **(e)** Quantification of frequencies from 2 independent experiments as mean ± SEM, *Il7r^wt/wt^ × Rorc^fl/fl^ × Tbx21^fl/fl^* n=6, *Il7r^cre/wt^ × Rorc^fl/fl^ × Tbx21^fl/fl^* n=7, Mann-Whitney-U testing. **(g)** Quantification of frequencies from 2 independent experiments as mean ± SEM, *Il7r^wt/wt^ × Rorc^fl/fl^ × Tbx21^fl/fl^* n=9, *Il7r^cre/wt^ × Rorc^fl/fl^ × Tbx21^fl/fl^* n=7, *Il7r^cre/wt^ × Rorc^fl/fl^ × Tbx21^fl/fl^ × Rora^fl/fl^* n=6, Kruskal-Wallis testing with Dunn’s multiple correction. Detailed statistics are available in source data.

As we observed residual Rorc transcriptional activity as well as GFP^lo/hi^ expression in both RKO and DKO ILCs, respectively, we further validated transcript and protein expression of RORγt in *Rorc(gt)^GFP/GFP^* and *Rorc(gt)^GFP/GFP^ × Tbx21^-/-^* mice, in which the ATG in front of *Rorc* exon 1γt is replaced by GFP gene insertion disrupting the transcriptional start site (TSS) (Fig. 8a). We confirmed absence of exon 1γt to 3 junction transcripts, while residual transcriptional activity of exon 5 to 6 junction could be detected in intestinal ILCs from both RKO and DKO mice (Extended Data fig. 8a and b), along with expression of a protein stained by commonly used anti-RORγ(t) antibodies (Extended Data fig. 8c). We also confirmed that *Rorc* exons 1 and 2 (encoding RORγ) were not transcribed in ILCs from DKO mice (Extended Data fig. 8a). Despite these surprising findings, thymic disruption of T cell development typically associated to RORγt deficiency^12,47,48^ was observed in RKO, and could not be rescued in DKO mice (Extended Data fig. 8d), indicating that the hypomorphic RORγt protein is not functional either in RKO or in DKO mice.

To rule out that LN formation observed in DKO mice was due to residual function of this truncated protein, we validated our findings by using alternative models of RORγt deficiency (Fig. 8a). To this aim, we crossed *Rorc(gt)^Δex4^* mice, which carry a conventional deletion in exon 4 of the *Rorc* locus, leading to disruption of the DNA-binding domain (DBD) of RORγt (Fig. 8a) to *Tbx21^-/-^* mice. Moreover, we took advantage of *Rorc(gt)^fl/fl^* mice, in which *Rorc* exons 3 to 6 are floxed, and cre recombination leads to deletion not only of the DBD but also of the hinge and ligand-binding domain (LBD) regions of RORγt (Fig. 8a). *Rorc(gt)^fl/fl^* mice were crossed to *Tbx21^fl/fl^* and *II7r^cre/wt^* mouse lines enabling conditional deletion of T-bet as well as RORγt exon 3-6 in IL-7R^+^ lymphocytes, including ILC progenitors. Importantly, both additional models of concomitant RORγt and T-bet deletion phenocopied *Rorc(gt)^GFP/GFP^ × Tbx21^-/-^* mice, resulting in presence of LN (Fig. 8b,c), which were absent in the RORγt single KO counterparts (*Rorc(gt)^Δex4^* and *II7r^cre/wt^ × Rorc(gt)^fl/fl^* mice) (Figure 8B and C). Thymic T cell development was impaired in both additional models (Extended Data fig. 8d) further corroborating the absence of functional RORγt protein. In support of this, no protein staining could be detected in *Il7r*cre/wt x *Rorc(gt)^fl/fl^ × Tbx21^fl/fl^* mice using the same anti-RORγt antibody (Extended Data fig. 8e). PLZF^hi^ ILCP were enriched in mLN of both additional mouse strains lacking RORγt and T-bet (Fig. 8d,e), reminiscent of our observations in DKO versus reporter mice. Thereby, our data indicate that, in the absence of T-bet, LN development and accumulation of PLZF^hi^ ILCP with LTi signatures occurs in the absence of RORγt TSS ATG, DBD, hinge and LBD regions, demonstrating that acquisition of LTi functions, but not full ILC3 maturation or thymic T cell development, can happen independently of RORγt.

Finally, to assess whether other transcription factors could promote acquisition of LTi signatures and LN development in the absence of RORγt and Tbet, we analysed the role of RORα, which cooperatively regulates Th17 genes together with RORγt^49–51^. *RORa* was highly expressed in ILCP from all mouse strains (Figure S8F) and its additional deletion, as shown by crossing *Il7r^cre/wt^* × *Rorc(gt)^fl/fl^* × *Tbx21^fl/fl^* mice with *Rora^fl/fl^* mice, resulted in complete lack of LN (Figure 8F), as well as of intestinal PLZF^hi^ ILCP (Fig. 8g).

Altogether, our data unravel a complex interplay between T-bet, RORγt and RORα in regulating fetal ILCP differentiation, with dramatic impact on LN development during embryonic life.

## Discussion

ILCs are among the first lymphocytes to appear during ontogeny to seed organs and tissues. While adult intestinal ILCs have been investigated also at a single cell level^23,52^, our scRNA-seq analysis of LinLD^-^CD127^+^ and/or CD122^+^ cells from fetal intestine enabled us identifying the entire spectrum of mature ILCs, including ILC3, ILC2 as well as a consistent pool of ILC1 residing at this site, and characterizing their global transcriptional signatures. Besides mature ILC subsets, the embryonic intestine also holds a range of hematopoietic progenitors, with signatures compatible with the ones observed in CLP and ILCP derived from FL or adult BM. By using Arg1 reporter mice, Locksley and colleagues have previously described a population of Arg1^+^ ILCP in fetal intestine^53^. As the ILCP cluster emerged in our scRNA-seq data set is highly enriched in Arg1 transcripts, these progenitors might largely overlap. CLP and ILCP populations have also been recently described in peripheral LN anlagen at E13.5 and E14.5^54^, suggesting that FL CLP and ILCP might seed these sites and differentiate *in situ* during embryonic development.

Remarkably, we detected certain heterogeneity within the transcriptome of embryonic E18.5 intestinal ILC3, which does not follow the canonical separation between LTi_0_ and LTi_4_, as CD4 expression does not resolve clustering of different fetal ILC3 subsets. Most strikingly, we observed the appearance of an unappreciated T-bet^+^ ILC3 subset before birth. It was previously shown that NKp46^+^T-bet^+^ ILC3 emerge postnatally with colonization of the intestine by microbiota^14^. While our data confirm the prenatal absence of ILC3 expressing NKp46 protein, our T-bet analyses evidently demonstrate that ILC3 expression of T-bet transcripts and protein starts already as early as E14.5 during embryonic development. Interestingly, we additionally found a T-bet^+^RORγt^+^CD4^+^ population displaying some phenotypic similarities with classical LTi cells, which is virtually absent in the adult intestine. Together with the observed asynchronous expression of CCR6 and CD4 on fetal ILC3 populations, these data indicate that the embryonic intestine already holds remarkable ILC3 heterogeneity, but showcases a different spectrum of ILC3 subsets as compared to adult intestine, possibly suggesting diverse waves of ILC3 differentiation. Contrary to the prevailing view, our data indicate that acquisition of T-bet and generation of plastic transcriptional states by loss of RORγt or T-bet can occur already prenatally and might result from cellular interactions or local cytokine milieus in the developing tissue.

With the intention to delineate the role of T-bet in ILC3 lineage specification during embryogenesis, we observed that lack of T-bet restored development of peripheral LN, typically absent in RORγt-deficient mice. This phenotype could be observed in three independent mouse models, in which the *Rorc* locus was differentially disrupted. Notably, in *Rorc(gt)^GFP/GFP^ × Tbx21^-/-^* and to a lower frequency in *Rorc(gt)^GFP/GFP^* mice (the latter being the most commonly used model of RORγt deletion for immunological studies in the last fifteen years), we observed the presence of intestinal ILCs displaying residual low to high expression of GFP as well as of a protein reacting with commercially available antibodies, whose specific epitope however has not been characterized. Although the significance and function of this protein remains to be determined, in our study we could exclude that it plays a role in LN restoration, as the same phenotype could also be observed in *II7r^cre/wt^* x *Rorc(gt)^fl/fl^* × *Tbx21^fl/fl^* mice. In these mice, all functionally relevant regions for RORγt activity, namely the DBD, hinge and LBD, are deleted in *Il7r*^+^ cells and no residual protein could be detected. Notably, LN formation in all double knockout strains was not associated with the appearance of ILC3, but rather with the accumulation of an ILCP population expressing PLZF. Numbers of PLZF^hi^ PD1^hi^ ILCP were increased in DKO as compared to reporter mice, in which ILCP might continuously differentiate towards the mature ILC lineages. Accumulation of PLZF^hi^ ILCP in the absence of RORγt and T-bet was not linked to increased proliferation, but might possibly be sustained by the anti-apoptotic molecule Bcl2, whose transcript expression was significantly elevated in ILCP from DKO mice.

By using *Zbtb16^GFPcre^* FM mice, It was previously shown that PLZF transiently marks a FL and BM ILCP, which has lost LTi potential, while mature ILCs are largely negative for PLZF^33^. Our data confirm high enrichment of *Zbtb16* transcripts also in ILCP from embryonic intestine and mLN anlage^55^, but demonstrate that *Zbtb16* expression is also present at a lower level in fetal ILCs especially within ILC3/LTi cells, in line with a recent report by McKenzie et al.^56^. Notably, PLZF^hi^ ILCP in DKO mice are enriched in cells expressing important LTi molecules, such as *CXCR5*, RANKL and Ltα_1_β_2_. Expression of these molecules was previously described on FL and BM-derived PLZF^hi^ ILCP and seems therefore to be induced at an early stage of ILCP differentiation^1,57^, possibly when transient expression of mixed lineage-specific transcriptional patterns occurs^56,57^. These data suggest that RORγt is important to drive full differentiation of the ILCP towards the ILC3 lineage, but seems redundant for the acquisition of central LTi molecules and related functions, such as LN development. However, LTi functions of these progenitors only manifest in the absence of T-bet and do not emerge in RORγt single deficient mice, where ILCP preferentially differentiate towards ILC1 (and ILC2). These data indicate that a major role for RORγt is to counteract T-bet^-^driven differentiation of fetal ILCP towards ILC1 (and NKp46^+^ ILC3), highlighting that the balance between these two transcription factors regulates not only ILC3-ILC1 lineage fate postnatally, but also prenatally. Our data imply that other TFs compensate for RORγt and promote expression of LTi molecules. Indeed, we could show that additional deletion of RORα, a ROR-family TF which cooperatively regulates Th17 genes together with RORγt^49–51^, abolished LN development as well as accumulation of PLZF^hi^ ILCP. Accordingly, the study by Withers and colleagues^58^ (in-press NI 2021) reports a role for RORα in supporting postnatal intestinal ILC3 functions, specifically IL-22 expression, in mice lacking RORγt and T-bet and observes a similar role for RORγt in counteracting T-bet^-^driven differentiation of mature ILC3.

Our observations unveil a new role for T-bet in embryogenesis and demonstrate that a strict balance of RORγt and T-bet in differentiating ILCP governs the ILC3-ILC1 equilibrium, while RORα sustains PLZF^hi^ ILCP accumulation and functions before and after birth.

## Methods

### Mice

*Tbx21-ZsGreen* mice 59, *Rorc(gt)^GFP/wt^* ^13^, *Rorc(gt)^Δex4^* ^12^, *Tbx21^-/-^* ^60^ and Rag2^-/-^^61^ were used in this study. *Rorc(gt)^cre^* ^62^ and *Tbx21^cre^* ^63^ mice were crossed to *R26R^eYFP^* and used for *Rorc(gt)*- or *Tbx21*-fate mapping studies. *Rorc(gt)^cre^* was carried only by female breeders to prevent germline YFP expression. To obtain mice with specific deletion of *Rorc* and/or *Tbx21* and *Rora* in *II7r*-expressing cells, *Rorc^fl/fl^* ^64^ and/or *Tbx21^fl/fl^*(purchased from Jackson, stock ID 022741) and *RORa^fl/fl^* ^65^ were crossed to *II7r^cre^* ^66^ mice in cooperation with David Withers, Birmingham. *Rag2^-/-^ll2rg^-/-^* mice were crossed to express the CD45.1 in our facility. Timed pregnancies were accomplished by mating in the evening; evidenced copulation was checked in the morning and females then considered E0.5. All mice were bred under specific pathogen-free conditions in the animal facility of the Federal Institute for Risk Assessment (Berlin, Germany) and the Research Institute for Experimental Medicine (FEM) of the Charité (Berlin, Germany). Animal handling and experiments were conducted according to the German animal protection laws and approved by the responsible governmental authority (Landesamt für Gesundheit und Soziales).

### Tissue dissociation

Single cell suspensions from intestinal lamina propria of adult animals were obtained as described before^67^. In short, intestines were isolated and PPs were removed. Tissue was cleaned, longitudinally opened and chopped into pieces of 2 cm, followed by two rounds à 15 min at 37°C of epithelial cell dissociation using Hank’s balanced salt solution without calcium and magnesium (HBSS-/-, Gibco) supplemented with 2% FCS, 10 mM HEPES buffer pH 7,5 (Sigma), 1 mM DTT (ThermoFisher) and 5 mM EDTA (Sigma). After washing with HBSS-/-, tissues were digested using a lamina propria dissociation kit (Miltenyi) according to the manufacturer’s instructions. Lymphocytes were further enriched on a 40%/80% Percoll (GE Healthcare) gradient.

Small intestines from fetal mice were isolated from embryos under a dissecting microscope. Surrounding mesenteric tissue was removed under further magnification and cut open longitudinally. Tissue was incubated for 30 min at 37°C under agitation in HBSS containing calcium and magnesium (HBSS^+^/^+^), 2% FCS, 10 mM HEPES, 0.2 U/mL Liberase DL (Sigma) and 50 μg/mL DNasel (Sigma). Leftover tissue was dissociated using a 22G needle and cell suspension was passed through a 70 μm strainer and washed in PBS/BSA. E14.5 and E16.5 time points were pooled samples; E18.5 intestines were treated separately.

Fetal liver was isolated under a dissecting microscope and a single cell suspension was generated by vigorously pipetting using a 1000 μL pipette. Cell suspension was filtered through a 70 μm strainer, washed in PBS/BSA followed by erythrocyte lysis.

Embryonic lymph node anlagen from the mesenteric region were dissected under a microscope or isolated from adult animals and passed through a 70 μm strainer.

### Flow cytometry

Cells were stained using the following antibodies: APC-Vio770 anti-mouse CD19 (Miltenyi, REA749, 1:50), APC-Fire780 anti-mouse F4/80 (BioLegend, BM8, 1:100), APC-Fire780 anti-mouse Gr-1 (BioLegend, RB6-8C5, 1:200), APC-Cy7 anti-mouse FcεRIα (BioLegend, MAR-1, 1:100), BV510 (BioLegend, 1:200) or BUV396 (BD, 1:200) anti-mouse CD45 (30-F11), APC anti-mouse CD45.1 (Miltenyi, A20, 1:10), VioGreen anti-mouse CD45.2 (Miltenyi, 104-2, 1:10), PerCP-eF710 anti-mouse γδTCR (ThermoFisher, GL-3, 1:100), A700 (BioLegend, 1:100) or PerCP-Cy5.5 (BioLegend, 1:100) anti-mouse βTCR (H57-597), PerCP-Vio700 anti-mouse CD3 (Miltenyi, REA641, 1:30), BV605, PE-Dazzle594 or PE (all BioLegend 1:50) anti-mouse NKp46 (29A1.4), BV650 (1:200), BV711 (1:100), PE-Cy7 (1:400), BV785 (1:100) or AlexaFlour647 (1:400) anti-mouse CD4 (all BioLegend, RM4-5), BV650 or BV711 anti-mouse CD8 (all BioLegend, 53-6.72, 1:200), BV785 anti-mouse CD127 (BioLegend, A7R34, 1:100), PE-Vio770 anti-mouse CD122 (Miltenyi, REA1015, 1:50), PE anti-mouse CCR6 (BioLegend, 29-2L17, 1:300), PE-Vio615 anti-mouse CXCR5 (Miltenyi, REA215, 1:10), PerCP-eF710 anti-mouse CXCR6 (ThermoFisher, DANId2, 1:200), Alexa700 anti-mouse CD90 (in-house production, T24, 1:600), BV421 anti-mouse α_4_β_7_ (BD, DATK32, 1:150), PE-Cy7 (1:300) or BV605 (1:200) anti-mouse KLRG1 (BioLegend, 2F1/KLRG1), BUV661 (BD, 1:100) or BV785 (BioLegend, 1:200) anti-mouse NK1.1 (PK136), PE-Vio770 anti-human/rat/mouse ICOS (Miltenyi, REA192, 1:10), BUV563 anti-mouse CD38 (BD, 90/CD38, 1:100), BV711 anti-mouse IgD (BioLegend, 11-26c.2a, 1:100), PerCP-eF710 anti-mouse GL7 (ThermoFisher, GL7, 1:100), PE anti-mouse Fas (BD, Jo2, 1:400), BV510 (1:100) or BV711 (1:200) or PE-Cy7 (1:200) anti-mouse PD1 (all BioLegend, 29F.1A12). For staining with PE anti-mouse RANKL (BioLegend, IK22/5, 1:50) and LTα_1_β_2_-hFab^68^ (1:100) and anti-hIgG APC (Miltenyi, IS11-12E4.23.20, 1:50) cells were incubated for 1 h at 37°C in complete RPMI medium containing 10% FCS. To minimize non-specific binding of antibodies, cells were blocked with anti-mouse CD16/32 (in-house production, 2.4G2, 1:100) and dead cells were excluded by staining with Fixable Viability Dye (LD) (eBioscience, 1:5000) prior to staining. For intranuclear staining, cells were fixed with transcription factor staining buffer set (BD Bioscience) according to the manufacturer’s instructions. For fixation of fate-map YFP signal, cells were fixed 20 min at 25°C in 2% para-formaldehyde (EMS) and stained in BD Bioscience perm buffer with PE-Vio615 (1:10) or FITC (1:10) anti mouse/human GATA3 (Miltenyi, REA174), AlexaFluor647 anti-mouse T-bet (BioLegend, 4B10, 1:500), PE (1:800) or BV650 (1:200) or BV421 (1:150) anti-mouse RORγt (BD, Q31-378), eFluor450 Eomes (ThermoFisher, Dan11mag, 1:200), AlexaFluor647 anti-mouse/human PLZF (BD, R17-809, 1:800), APC antimouse Bcl-6 (Miltenyi, REA373, 1:50), A700 anti-mouse Ki67 (BioLegend, 16A8, 1:100). Unless otherwise stated all antibodies were purchased at Miltenyi, Themofisher, Biolegend UK or BD Biosciences.

For the assessment of IL-22 and IL17A-producing cells, single-cell suspensions obtained from small intestinal lamina propria were stimulated in 10% FCS IMDM and 10 ng/mL of PMA (Sigma-Aldrich) and 500 ng/mL ionomycin (Sigma-Aldrich) in the presence of GolgiPlug containing brefeldin A (BD Biosciences) for 4 h at 37°C. Following washing, cells were surface labelled with above listed antibodies and fixed in 1x InsideFix (Inside Stain Kit Miltenyi Biotec) for 20 min at 25°C. Intracellular staining using APC anti-mouse IL-22 (ThermoFisher, IL22JOP, 1:100) and BV711 anti-mouse IL-17A (BioLegend, TCC11-18H10.1, 1:100) was performed in InsidePerm (inside Stain Kit Miltenyi Biotec) for 30 min at 25°C.

Flow cytometry was performed using a LSR Fortessa and BD FACSymphony flow cytometer (BD Biosciences). Data were further analysed with FlowJo Software v10 (Flow Jo). All flow cytometry analysis was performed according to the Guidelines for the use of flow cytometry and cell sorting in immunological studies^69^.

### *in vitro* cell culture

Single-cell suspensions from E14.5 fetal liver were enriched for CD45^+^ cells using magnetic activated cell sorting, followed by staining with fluorochrome-conjugated antibodies and sorted on BD FACSAria II (BD Bioscience) as live Lin-CD45^+^Flt3^-^CD127^+^α_4_β_7_^+^PD1^-^ cells. Sorted lymphocytes were subsequently resuspended in IMDM (Gibco BRL Life Technologies) supplemented with 10% FCS, 100 U/mL penicillin, 0.1 mg/mL streptomycin, 50 μM β-mercaptoethanol (Gibco BRL Life Technologies) and each 20 ng/mL recombinant mouse IL-7 and SCF. Cells were plated onto 70% confluent OP9 stromal cells in round bottom 96-well plates and medium was renewed every three days by replacing half of the media. Cells were analysed by flow cytometry after 5-8 days of culture.

### Adoptive transfer

For adoptive transfer experiments, *Rag2^-/-^II2rg^-/-^* CD45.1 recipient mice (Thy1.2/1.2) were sub-lethally irradiated with 5 Gy and endogenous, irradiation-resistant ILC3 populations were furthermore depleted by i.p. administration of 250 μg anti-mouse Thy1.2 (30H12, in-house production) every third day. E14.5 FL lymphoid progenitors were FACS-sorted on an Ariall (BD Biosciences) instrument using the following sorting strategy LinLD^-^ CD45^+^CD127^+^. CD45.2^+^ donor cells (Thy1.1/1.1 congenic) were injected i.v. Mice were kept under antibiotic treatment in the first two weeks and analysed 4 weeks after transfer.

### Lymph node enumeration

Percentages of mice with LN were calculated for each LN type using the total number of mice with the respective LN found in a given strain relative to the maximal number of mice with LNs of that type found in *Rorc(gt)^GFP/wt^* or *Il7r^cre^* control mice.

### Immunofluorescence staining

Lymph nodes were fixed for 4 h in 4% paraformaldehyde and dehydrated via a sucrose gradient for 24 h, embedded in O.C.T. compound and frozen in 2-Methylbutane within ethanol rinsed dry ice bath. Frozen lymph nodes were cryotome-cut into 7 μm sections and placed on microscope slides in pairs. After thawing and PBS-rehydration of slides, primary staining was carried out after 20 min blocking with 10% goat serum in PBS/BSA-0.1%Tween20 (P-B-T). DAPI, anti-igD-AF594, PNA-bio and rat-anti-mouse-CD35 were incubated for 1 h in P-B-T. Secondary staining was performed after washing in P-B-T by 30 min incubation with SA-A555 and anti-rat-IgG-A647 in P-B-T. Samples were finally washed in PBS and covered with mounting medium and cover slip and before image acquisition was performed on Zeiss LSM 880 confocal microscope.

### Quantitative real-time PCR

mRNA was isolated from small intestinal intraepithelial cells by using NucleoSpin® RNA (Macherey-Nagel) and following the manufacturer’s protocol. RNA integrity was assessed on a BioAnalyzer (Agilent) and RNA samples with a RIN score ≥ 7 were further transcribed into first strand cDNA by using Reverse Transcription Reagents (Applied Biosystems). qPCR reactions were assayed in triplicates per sample by using a StepOne Plus real-time PCR system and TaqMan Gene expression assays (all Thermofisher): *Reg3g* (Mm00441127_m1), *Reg3g* (Mm00440616_g1), *Rorc1-3* (Mm03682796_m1), *Rorc5-6* (Mm01261021_g1), Gapdh (Mm99999915_g1), *Hprt* (Mm03024075_m1) and *Actb* (Mm02619580_g1). mRNA content was normalized relative to the mean expression of the arithmetic means of Gapdh, *Hprt* and *Actb* CT values by applying the comparative C_T_ method (2 ^-ΔCT^) in which ΔC_T (gene of interest)_ = C_T (gene of interest)_ - C_T (arithmethic mean of housekeeping reference value)_.

### RNA sequencing and data analysis

RNA of cells was extracted using RNeasy Plus Micro Kit (Qiagen) from sorted small intestinal lamina propria cells using the following markers LinLD^-^ CD45^+^CD3-RORγt-GFP^+^ expressing either NKp46 or CD4. RNA quality was assessed by an Agilent 2100 Bioanalyzer. SMART-SeqII (ultra-low input RNA) libraries were prepared using Nextera XT DNA sample preparation kit (Illumina). Sequencing was performed on an Illumina HiSeq4000 platform, PE100. For heatmaps, normalized RNA-seq data z-score transformed were plotted using ggplot2 R package. RNA-seq datasets analysed are publicly available in Gene Expression Omnibus repository with the accession number GSE161439.

### 10x Genomics Chromium single-cell RNA-sequencing

Fetal small intestinal lymphocytes were isolated as described above and sorted as LinLD^-^CD45^+^CD122^+^ and/or CD127^+^. A total of 7 embryos from one pregnant *Rorc(gt)^GFP/wt^* female, 18 embryos from two pregnant *Rorc(gt)^GFP/GFP^* females and 19 embryos from three pregnant *Rorc(gt)^GFP/wt^ × Tbx21^-/-^* females were used. Samples were furthermore labelled with antibody-derived tags against specific extra-cellular targets and genotypes hash-tagged with TotalSeq™-A antibodies (BioLegend) following the manufacturer’s protocol. A total of 8000 cells were loaded in the Chromium™ Controller for partitioning single cells into nanoliter-scale Gel Bead-In-Emulsions (GEMs). Single Cell 3’ reagent kit v3.1 was used for reverse transcription, cDNA amplification and library construction of gene expression libraries (10x Genomics) according to the manufacturer’s instructions. TotalSeq™-A libraries were prepared following the protocol for 10x Single Cell 3’ Reagent Kit v3.1 provided by BioLegend, including primer sequences and reagent specifications. Concentrations of all libraries were quantified using a QubitTM 2.0 Fluorometer (ThermoFisher) and quality was tested on a 2100 Bioanalyzer with High Sensitivity DNA kit (Agilent). Sequencing was performed using the NextSeq500/550 HighOutput Kit v2.5 (150 cycles) on a NextSeq500 sequencer (Illumina). scRNA-seq datasets analysed are publicly available in Gene Expression Omnibus repository with the accession number GSE161441.

### scRNA-seq data processing

Demultiplexing and alignment to the mm10 reference transcriptome was performed with Cell Ranger v3.0.2^70^. Antibody- and hashtag-barcodes were counted with CITE-seq-Count^71^ and normalized by centred log ratio transformation with Seurat v3.0.2^72^, which was also used for further data processing. Genotypes were assigned based on normalized hashtag counts. Cells were filtered by number of transcripts, genes, percent mitochondrial genes and for cells present within transcriptome, antibody and hashtag libraries. Normalized expression values were generated with scran v1.16.0^73^, followed by scaling and principal component (PC) analysis. PC loadings and explained variance were visualized to determine PCs used for dimensionality reduction by uniform manifold approximation (UMAP)^74^ and Seurat clustering. Wilcoxon test was applied for differential gene expression analysis of genes expressed by ≥10% of cells in a cluster and with a log fold change of ≥0.25. Lineage structures on UMAPs were analysed with Slingshot v 1.4.0^75^, giving the starting (CLP) and end populations (ILC1/2/3) as input. Partition-based graph abstraction (PAGA) using the Fruchterman & Reingold algorithm was performed within Scanpy v1.4.6 ^76^ after import of preprocessed Seurat objects into Python with anndata2ri^77^. scRNA-seq data analysis was performed with R v4.0.0 and Python v3.7.6.

### Statistical analysis

No statistical method was used to pre-determine sample sizes and no randomization was performed. Data collection and analysis were not performed blind to the conditions of the experiments. Kruskal-Wallis test with Dunn’s multiple comparison correction was employed for statistical analysis of datasets comparing three groups. Two-tailed Mann-Whitney test was performed for statistical analysis of datasets where two groups were compared. Statistical analyses were performed with Prism 5.04 (GraphPad Software) using a confidence level of 0.95, and P values >0.05 were considered not significant, P ≤ 0.05 were considered as *, P ≤ 0.01 were considered as **, P ≤ 0.001 were considered as ***, P ≤ 0.0001 were considered as ****

## Extended Data

## Data Availability

Raw transcriptome data reported in this paper are deposited and available from the Gene Expression Omnibus under accession code GSE161441 (scRNA-seq) and GSE161439 (bulk RNA-seq). Sequencing data were aligned to the mm10 reference transcriptome. Source data are provided with this paper. All other data supporting the findings of this study are available within the paper or from the corresponding author upon request.
